# *Corylus avellana* L. Natural Signature: Chiral Recognition of Selected Informative Components in the Volatilome of High-Quality Hazelnuts

**DOI:** 10.3389/fpls.2022.844711

**Published:** 2022-04-25

**Authors:** Federico Stilo, Marta Cialiè Rosso, Simone Squara, Carlo Bicchi, Chiara Cordero, Cecilia Cagliero

**Affiliations:** ^1^Dipartimento di Scienza e Tecnologia del Farmaco, Università degli Studi di Torino, Turin, Italy; ^2^Laemmegroup S.r.l - A Tentamus Company, Moncalieri, Italy

**Keywords:** *Corylus avellana* L. hazelnut fruits, chiral natural signature, native chiral volatiles, key-aroma compounds, hazelnuts volatilome

## Abstract

The volatile fraction of plant-based foods provides useful functional information concerning sample-related variables such as plant genotype and phenotype expression, pedoclimatic and harvest conditions, transformation/processing technologies, and can be informative about the sensory quality. In this respect, the enantiomeric recognition of the chiral compounds increases the level of information in profiling studies, being the biosynthesis of native compounds often stereo-guided. Chiral native volatiles mostly show an enantiomeric excess that enables origin authentication or support correlation studies between chemical patterns and sensory profiles. This study focuses, for the first time, on the enantiomeric composition of a large set of chiral compounds within the complex volatilome of *Corylus avellana* L. belonging to different cultivars (*Tonda Gentile Romana*, *Tonda Gentile Trilobata*, *Anakliuri*) and harvested in different geographical areas (Italian and Georgian). Besides native components profiled in raw kernels, volatiles formed after technological treatment (i.e., roasting) are also considered. Headspace solid-phase microextraction combined with enantioselective gas chromatography–mass spectrometry enables the accurate tracking and annotation of about 150 compounds across many samples. The results show that chiral compounds have diagnostic distribution patterns within hazelnut volatilome with cultivar and harvest region playing the major role. Moreover, being some of these chiral molecules also key-aromas, their distribution has a decisive impact on the sensory properties of the product. In particular, the enantiomeric composition of (*E*)-5-methyl-2-hepten-4-one (filbertone) resulted to be discriminant for origin authentication. The enantiomeric distribution showed, according to literature, an excess of the (*S*)-enantiomer in both raw and roasted samples volatilome with larger differences in raw samples. The amount of both (*R*) and (*S*)-filbertone increases during roasting; the most marked increase for (*R*)-enantiomer is observed in Italian samples, thus supporting evidence of better hedonic properties and more pleasant odor and aroma.

## Introduction

The volatile fraction of a plant, also referred to as *volatilome* (Maffei et al., 2011; Bicchi and Maffei, 2012), is characterized by a complex mixture of compounds belonging to different chemical classes. These volatiles, mainly terpenoids, fatty acid degradation products, phenylpropanoids, and amino acid-derived products, show molecular weights generally below 350 Da, are characterized by medium-to-high log K_o/w_, and are readily released or vaporized from the condensed phase (solid or liquid) under suitable conditions (temperature, pressure, solubility in the medium, ion strength, etc.). They play a fundamental role in defending against herbivores and pathogens, attracting pollinators and seed dispersers, and acting as signals in plant-plant communication (Cagliero et al., 2021b). Besides their role for the plant, volatiles play a wide range of biological activities for humans. In particular, in the food field, volatiles confer typical aroma properties and their study can be informative about the sensory quality of a given product (Dunkel et al., 2014; Cordero et al., 2015, 2019). The volatile fraction of foods also provides useful functional information concerning sample-related variables such as plant genotype and phenotype expression, pedoclimatic and harvest conditions, postharvest processing and treatments, shelf-life storage conditions, and the effects of transformation/processing technologies (Cialiè Rosso et al., 2018; Sgorbini et al., 2019a).

In addition, it is important to consider that the biosynthesis of some key odorants is very often stereo-guided and usually results in chiral components with a more or less marked enantiomeric excess. At the same time, the interaction of the odorants with olfactory receptors is mainly stereoselective, meaning that the enantiomers of a chiral molecule might present different organoleptic properties while eliciting different odor sensations and/or intensities (Koppenhoefer et al., 1994; Brenna et al., 2003; Cagliero et al., 2017).

Enantiomeric recognition of chiral components and enantiomeric excess and/or ratio determinations are therefore important aspects not only to correlate chemical patterns to the sensory profile of food, but also to authenticate geographical origin for quality assurance purposes. Moreover, enantiomeric recognition enables conformity monitoring vs. legal requirements while objectively supporting fraud/adulteration counteractions (Marchelli et al., 1996; Cagliero et al., 2016; Langen et al., 2016; Zhu et al., 2017; Sgorbini et al., 2019b).

European hazelnut (*Corylus avellana* L.) is a diploid (2*n* = 2*x* = 22), monoecious, open-pollinated tree. It is one of the most cultivated nut crops worldwide. This species spreads from Asia Minor and Caucasus regions to Europe and North Africa; it includes several cultivars, biotypes, and accessions that show a high level of genetic diversity for traits such as vigor, growth habits, suckering, nut size and shape, and shell thickness. In particular, hazelnut kernels have a relevant role in agroindustry due to their nutritional, unique, and distinctive flavor which makes them appreciated as an ingredient in a variety of dairy, bakery, confectionery, candy, and chocolate products. A minor part of the nut crop is consumed as such, whereas the major part of the harvest undergoes the roasting process. Roasting improves its color, crunchy, and crispy texture and enriches the flavor of *burnt*, *coffee-chocolate-like*, and *roasty* notes thanks to the formation of odor active compounds belonging to ketones, aldehydes, acids, alcohols, heterocycles (pyrazines, furans, pyrroles), and aromatics (Nicolotti et al., 2013a,b; Göncüoğlu Taş and Gökmen, 2017; Marzocchi et al., 2017; Taş and Gökmen, 2019).

Hazelnuts market standards imply severe quality control procedures to comply with the desirable hedonic profile of finished products. The cultivar, cultural techniques, geographical origin, harvesting time, post-harvest management and processing, shelf-life storage conditions, and morphological aspect are the major variables influencing the phytochemical profile of the nuts and, thereby, their aroma, which makes them the main parameters monitored in the final hazelnut quality assessment (Locatelli et al., 2011; Ciarmiello et al., 2014; Klockmann et al., 2017; Pedrotti et al., 2021).

The hazelnut volatile fraction has been widely investigated in particular in sensomic studies aiming to correlate the odorants pattern of raw and roasted samples to their aroma (Alasalvar et al., 2003a; Burdack-Freitag and Schieberle, 2010, 2012; Kiefl et al., 2012, 2013; Pedrotti et al., 2021) and also to discriminate between different cultivars and/or geographical origins (Alasalvar et al., 2006; Cordero et al., 2008, 2010; Kiefl and Schieberle, 2013).

However, information is lacking concerning the enantiomeric recognition of many known aroma compounds. In this regard, the attention has been mainly focused on the enantiomeric characterization of the hazelnut’s key-odorant, (*E*)-5-methyl-2-hepten-4-one (i.e., filbertone), and of its geometrical isomer, (*Z*)-5-methyl-2-hepten-4-one, since the four diastereoisomers are characterized by different sensory properties (Güntert et al., 1991; Puchl’ová and Szolcsányi, 2018). Güntert et al. (1991) performed a full assessment and olfactory comparison of all four possible stereoisomers showing that the (+)-(5*S*)-(*E*)-5-methyl-2-hepten-4-one has *hazelnut*, *fatty*, *metallic*, *balsamic* notes, the (-)-(5*R*)-(*E*)- and (-)-(5*R*)-(*Z*)-isomers show *hazelnut* and *woody* odors, and the (+)-(5*S*)-(*Z*)-5-methyl-2-hepten-4-one has *hazelnut*, *woody, fatty, metallic* notes. In addition, the odor threshold of (*S*)-(E)-filbertone has been estimated as 10-fold lower than the (*R*)-(E)-enantiomer and threefold lower than the (+)-(*S*)-(*Z*)-5-methyl-2-hepten-4-one (Güntert et al., 1991). In the same study, the flavor perception of the two enantiomers of filbertone was evaluated showing a stronger *metallic, fatty, pyridine* impact for the (S)-enantiomer and a weaker *soft, butter, chocolate* impact for the (*R*)-enantiomer (Güntert et al., 1991).

Naturally occurring filbertone in hazelnuts exhibits low-to-medium enantiomeric excess, with a higher amount of *(S)*-enantiomer compared to the opposite enantiomer. The ratio between the two enantiomers is variable and depends on the origin of the hazelnuts, thermal treatment, analytical sample collection, and technological processing (Jauch et al., 1989; Güntert et al., 1991; Puchl’ová and Szolcsányi, 2018). There is also a remarkable difference between the enantiomeric excess of raw and roasted hazelnuts since, although the absolute amount of filbertone increases about 10-fold in roasted hazelnut compared to raw hazelnut (Pfnuer et al., 1999), the relative difference between the two enantiomers drop-down to lower enantiomeric excess for the *(S)*-filbertone (Güntert et al., 1991; Blanch and Jauch, 1998; Puchl’ová and Szolcsányi, 2018). Very interestingly, the racemization of *(S)*-filbertone is observed only after the thermal treatment of the kernels, while it does not occur when the pure standard compound is submitted to high temperatures, thus indicating that a precursor in hazelnut, hitherto not known, may form racemic filbertone by a non-stereoselective mechanism, thus increasing the relative amount of *(R)-*filbertone (Blanch and Jauch, 1998).

The aim of this study is therefore to evaluate the natural signature of native chiral volatiles and marker odorants in hazelnuts, focusing on the influence of functional variables, such as cultivar and harvest region (i.e., origin). Insights on the impact of the roasting process are also tackled by extending the profiling to newly formed components. The filbertone enantiomeric ratio is investigated to evaluate whether its chiral composition might be influenced by environmental factors, giving rise to a diagnostic chiral signature useful for authentication and quality assessment.

In this perspective, samples belonging to different cultivars and harvested in different geographical areas are here profiled to better understand the impact of external variables on the chiral natural signature. Headspace solid-phase microextraction (SPME) (HS-SPME) combined with enantioselective (ES) gas chromatography (GC) – mass spectrometry (MS) (ES-GC–MS) is adopted; the approach demonstrated good information potential and sampling reliability for quali/quantification and enantiomeric recognition of chiral analytes in food and plant-derived products (Cagliero et al., 2012; El-Dairi et al., 2012; Sgorbini et al., 2019b). Moreover, the headspace sampling limits the risk of racemization due to sample preparation, as already observed also for filbertone (Blanch and Jauch, 1998).

## Materials and Methods

### Hazelnut Samples

Hazelnut samples of industrial interest were from the 2017 harvest and characterized by homogeneous caliber (i.e., 13 mm) and suitable quality as confirmed by their disclosure after industrial quality control check. The samples were provided by Soremartec Italia Srl (Alba, CN Italy).

The samples’ selection was designed to cover:

• Cultivars: *Tonda Gentile Trilobata*—T is connoted by good attitudes for confectionery and has an excellent flavor profile, while *Anakliuri*—AN, a Georgian native cultivar, is of interest for its yields and good adaptation to specific pedo-climatic conditions. In addition, *Tonda Gentile Romana*—R, another Italian mono-cultivar was also analyzed to capture the enantiomeric distribution of some key odorants.
• Geographical area: each cultivar is harvested in its native territory (i.e., *Tonda Gentile Romana* in Italy—IT and *Anakliuri* in Georgia—GE), except for *Tonda Gentile Trilobata*, which was both harvested in Italy and implanted in Georgia to evaluate how pedoclimatic conditions would affect flavor potential.

Analyzed batches (*n* = 14) were obtained by industrial sampling protocols adopted for the safety and quality controls applied to all incoming raw materials. In particular, each sample was representative of a 1.00 tons batch; from each batch, three aliquots of 20 kg were further sampled and portioned (3 kg sub-aliquots) for grinding.

The analyses were conducted as 3 analytical replicates on finely grounded hazelnut powder obtained from the batch representative aliquots. Results reported in this study correspond to mean values of the 9 chromatogram profiles (*n* = 3 × 3) obtained for each incoming batch.

For Italian *Tonda Gentile Trilobata* (IT-T), Georgian *Tonda Gentile Trilobata* (GE-T), and Georgian *Anakliuri* (GE-AN), a total of 4 industrial batches/per origin were considered, while the Italian *Tonda Gentile Romana* (IT-R) was analyzed over 2 industrial batches.

### Reference Compounds and Solvents

Pure reference standards of (*E*)-5-methyl-2-hepten-4-one (filbertone), ethyl-2-methylbutyrate, α-pinene, linalool, and limonene used for identity confirmation were supplied by Merck (Milan, Italy). Working solutions were prepared in cyclohexane at a final concentration of 100 mg/L.

*n*-Alkanes (*n*-C9 to *n-*C25), adopted for linear retention indices (*I^T^*) calibration, were from Merck (Milan, Italy): the test mixture was prepared in cyclohexane at a final concentration of 100 mg/L.

### Volatiles Extraction by Headspace Solid-Phase Microextraction: Devices and Experimental Conditions

Headspace SPME sampling was done by the MPS-2 multipurpose auto-sampler by Gerstel GmbH & Co (Mülheim an der Ruhr, Germany). The extraction polymer was chosen according to previous studies (Cordero et al., 2008; Nicolotti et al., 2013a; Cialiè Rosso et al., 2018; Stilo et al., 2021) and consisted of a Divinylbenzene/Carboxen/Polydimethylsyloxane (DVB/CAR/PDMS) 50/30 μm film thickness—2 cm length from Merck (Milan, Italy). Before use, SPME fibers were conditioned at 270°C for 30 min as indicated by the manufacturer.

The hazelnuts were analyzed both raw and roasted. The roasting was carried out following a standardized lab-scale protocol (Cialiè Rosso et al., 2018; Rosso et al., 2021) in a laboratory ventilated oven on 100 g aliquots at 160°C for 15 min. Hazelnuts were then ground in a mortar in presence of liquid nitrogen to avoid over-heating and to keep the oily fraction in a condensed phase. The resulting powder, visually checked for uniform particle distribution, was then weighted up to 0.500 g in headspace vials of 20 ml volume. Sampling was carried out at 50°C under constant agitation (250 rpm) for 50 min.

### Enantioselective Gas Chromatography—Mass Spectrometry and Enantioselective Gas Chromatography-Flame Ionization Detector Platforms—Configuration and Settings

Gas chromatography-MS analyses were conducted on an Agilent 7890B gas chromatographic unit (Agilent Technologies, Wilmington, DE, United States) coupled with a single quadrupole MS Agilent 5977B equipped with a High-Efficiency Source (HES) and a high-frequency acquisition FID. The ionization voltage was set at 70 eV; the MS source was kept at 250°C and the MS quad at 150°C. The MS scan range was between 40 and 350 *m/z* with a scanning rate of 1,000 amu/s. The transfer line was set at 230°C and an HES-Tune option was used.

#### Chiral Stationary Phases Based on Cyclodextrin Derivatives

The following capillary GC columns were tested to select the best chiral selector for accurate profiling of volatiles and to assess the natural signature of selected chiral key-odorants: (a) 30% 2-*O*-methyl-3-*O*-methyl-6-*O*-*tert*-butyldimethylsilyl-β-cyclodextrin (MeMe-TBDMS β-CD) in PS-086; (b) 30% 2-*O*-acetyl-3-*O*- acetyl-6-*O*-*tert*-butyldimethylsilyl-β-cyclodextrin (AcAc- TBDMS-β-CD) in PS-086; (c) 30% 2-*O*-methyl-3-*O*-acetyl-6-*O*- *tert*-butyldimethylsilyl-β-cyclodextrin (MeAc-TBDMS-β-CD) in PS-086.

All columns’ dimensions were 25 m × 0.25 mm *d*_*c*_, 0.25 μm *d*_*f*_. and manufactured by MEGA S.r.l. (Legnano, Milan, Italy).

### Enantioselective Gas Chromatography Instrumental Settings

Solid-phase microextraction thermal desorption into the GC injector port was under the following conditions: split/splitless injector in pulsed splitless mode; pressure pulse of 35.67 kPa. The carrier gas was helium at a constant flow of 1.0 ml/min. The oven temperature program used with chiral selectors was chosen to ensure enantio-separation of the target chiral compounds and the higher resolution for (*E*)-5-methyl-2-hepten-4-one and was set as follows: from 40°C (1 min) to 180°C (4 min) at 2°C/min.

The *n-*alkanes liquid sample solution (100 mg/L each) for *I^T^* calibration and the standard reference solutions of chiral test and key-odorants were analyzed under the same temperature and flow rate programs and the following injection conditions: split/splitless injector in split mode, split ratio 1:50, injector temperature 240°C, injection volume 1 μl.

### Method Performance Parameters

Repeatability was evaluated on response data obtained by extracting selected *m/z* signals for targeted analytes. Percent (%) relative SD (RSD) was calculated across analytical replicates of a test sample (IT-R) analyzed every 2 days over the 2-week study (*n* = 21). The results are reported in Supplementary Table 1, the mean % RSD was 5.12% with maximum values for 2-phenyl-2-butenal (18.14%) and minimum for trimethyl pyrazine (0.03%).

### Data Acquisition and Data Processing

Data were acquired by Mass Hunter (Agilent Technologies, Wilmington, DE, United States) and processed by MSD ChemStation (Agilent Technologies, Wilmington, DE, United States). Statistical analysis and chemometrics were by XLSTAT statistical and data analysis solution (Addinsoft 2021, New York, United States), while heatmap visualization and Hierarchical Clustering (HC) were by Gene-E^1^ and Box-Plots by GraphPad Prism 9.3.0.463 (GraphPad Software, LLC, United States).

## Results and Discussion

This section was developed by following a rational workflow composed of different steps.

The first step included the selection of the more suitable and best performing chiral selector capable of separating the enantiomers of the chiral markers in hazelnuts, with a particular focus on (*E*)-5-methyl-2-hepten-4-one (or filbertone). Then, the composition of the volatile signature of raw and roasted hazelnuts was described, with insights on chiral compounds.

Data analysis was done by unsupervised and supervised methods to highlight the influence of the cultivar and harvest region on both, raw and roasted hazelnuts chiral volatilome. Moreover, the evolution of the volatiles pattern during roasting offered interesting insights on the formation/degradation pathways of the key components from their non-volatile precursors.

Finally, the added value of the chiral signature was explored by assessing the distribution of chiral compounds, with a particular emphasis on filbertone enantiomers.

### Selection of the Best Performing Chiral Selector

The first step of the study consisted of the selection of the best chiral selector capable of separating informative enantiomers in raw and roasted hazelnut volatilome, with a specific focus on filbertone. In particular, the chromatographic parameter adopted to guide the selection process was the resolution (*R*_*s*_) between the two enantiomers of filbertone and its isomer (*Z*)-5-methyl-2-hepten-4-one. Further performance evaluations were on other pairs of potent odorants (i.e., limonene, linalool, α-pinene, ethyl-2-methylbutyrate, γ-Pentalactone, γ-Hexalactone, γ-Heptalactone, γ-octalactone, γ-Nonalactone, and δ-Hexalactone). The three Enantioselective-GC (Es-GC) columns tested, selected on the basis of the existing literature, were: (a) MeMe-TBDMS-β-CD, (b) AcAc-TBDMS-β-CD (Liberto et al., 2008; Cagliero et al., 2021a), and (c) MeAc-TBDMS-β-CD.

Filbertone enantiomers were separated with a resolution of 9.00 by MeMe-TBDMS-β-CD; 7.65 with AcAc-TBDMS-β-CD; 14.34 with MeAc-TBDMS-β-C. Moreover, the asymmetric column (i.e., MeAc-TBDMS-β-C) was able to separate geometrical isomer (i.e., (*Z*)-5-methyl-2-hepten-4-one) and other targeted odorants of interest, thus resulting in the chiral selector of choice. Supplementary Table 2 lists the experimental *I^T^* and *R*_*s*_ for the chiral targeted volatiles above indicated, while Supplementary Figure 1 shows the chromatographic profiles obtained by analyzing the reference standards of 5-methyl-2-hepten-4-one four diastereoisomers.

### Capture of the Natural Signature of Hazelnuts Volatilome

The hazelnut’s volatilome is connoted by great complexity, including hundreds of detectable compounds belonging to different chemical classes (Cordero et al., 2010; Kiefl et al., 2012; Nicolotti et al., 2013a; Cialiè Rosso et al., 2018; Stilo et al., 2021). When the adopted analytical approach enables the further exploration of chemical dimensions, as in the case of ES-GC, the complexity and consequent chemical dimensionality increase (Giddings, 1995). Moreover, roasting promotes several chemical reactions on non-volatile primary metabolites resulting in the formation of new volatiles. On average, the number of detectable peaks, above a fixed signal-to-noise ratio (S/N) threshold value of 20, reached 100 for raw and 150 for roasted hazelnuts.

Supplementary Table 1 lists targeted analytes putatively identified in raw and roasted samples on the MeAc-TBDM-β-CD column, together with their experimental *I*^T^ and targeted ion (*m/z*) adopted for profiling purposes. The identification was by spectral similarity match above 95% (Agilent PBM algorithm) estimated over commercial and in-house databases, and *I*^T^ comparison with the in-house database of retention indexes when available (Liberto et al., 2008; Cagliero et al., 2017). Additional information on spectral data and *I*^T^s standard stationary phases can be retrieved in Supplementary Table 3 where chemical formulae and National Institute of Standard and Technology (NIST) database links are provided.

The elution order of the enantiomeric pairs of chiral compounds was used to assign the enantiomeric configuration: the *I*^T^s of unknown isomers were compared to those recorded in an in-house chiral library (Liberto et al., 2008) or with the *I*^T^ of reference standards of enantiomeric pure compounds, when available. For the chiral compounds for which data were not available about the enantiomeric elution order, the configuration was not assigned and they were denoted by (X) and (Y).

Table 1 lists potent odorants and key-aroma compounds together with their aroma quality, odor threshold (OT), and estimated odor activity values (OAVs) reported in the reference literature for raw and roasted *Tonda Gentile Romana* hazelnuts (Burdack-Freitag and Schieberle, 2010, 2012; Kiefl, 2013; Kiefl et al., 2013). As can be seen, many aroma compounds show a chiral center; the chirality of these molecules is conventionally not considered when evaluating their sensory properties, thus resulting in a single odor threshold and odor descriptor. However, as highlighted in Supplementary Table 4, the sensory properties of the two enantiomers of the main hazelnuts chiral aroma compounds and potent odorants, as well as their limits of odor detection, may substantially differ. The evaluation of the enantiomeric ratio of the chiral markers is therefore of fundamental importance to correlate the chemical composition of the hazelnut volatilome to their aroma profile.

**TABLE 1:** Potent odorants identified in raw and roasted Italian hazelnuts (cultivar *Tonda Gentile Romana*; Burdack-Freitag and Schieberle, 2010) with estimated OAVs according to Kiefl and Schieberle (Kiefl and Schieberle, 2013; Kiefl et al., 2013).

Compound	Symbol	Odor quality	Odor threshold in oil (mg/L)	OAV roasted	OAV raw
*3-Methylbutanal*	€	*Malty*	13	1,330	7
*2,3-Pentanedione*		*Buttery*	16	1,140	n.d.
*2-Acetyl-1-pyrroline*		*Popcorn-like*	0.1	360	2
*(Z)-2-Nonenal*	€	*Fatty*	4.1	300	1
*Dimethyl trisulfide*		*Sulfurous, cabbage*	0.01	164	n.d.
*2-Furfurylthiol*		*Coffee-like*	0.005 (in water)	86	n.d.
*2,3-Butanedione*	€	*Buttery*	10	85	n.d.
*4-Hydroxy-2,5-dimethyl-3 (2H)-furanone*	€	*Caramel*	60 (in water)	77	n.d.
*3-Methyl-4-heptanone*	€	*Fruity, nutty*		66	295
*3-(Methylthio)-propanal or methional*		*Cooked potato*	0.2	45	13
*2-Methylbutanal*	€	*Malty*	140	36	n.d.
*Octanal*		*Fatty,soapy*	56	28	3
*2-Thenylthiol*		*Coffee-like*	0.34	27	n.d.
*2-Ethyl-3,5-dimethylpyrazine*		*Earthy*	2	21	n.d.
*5-Methyl-(E)-2-hepten-4-one*	€	*Nutty, fruity*	8.7	13	2
*(E,Z)-2,4-Nonadienal*	€	*Deep-fried*	1.5	11	3
*(E,E)-2,4-Decadienal*	€	*Deep-fried*	166	10	n.d.
*Acetic acid*		*Sour*	114	8	3
*Hexanal*		*Green*	276	6	7
*(Z)-2-Octenal*	€	*Fatty*	50	2	n.d.
*2-Methoxyphenol*		*Phenolic, smoky*	15	2	n.d.
*3,5,5-Trimethyl-2 (5H)-furanone*	€	*Seasoning.-like*	3.1	2	n.d.
*2-Propionyl-1-pyrroline*		*Popcorn-like*	0.1	1	n.d.
5-Methyl-(Z)-2-hepten-4-one	€	*Nutty, fruity*	\	n.d.	n.d.
3-Methylbutanal	€	*Malty, almond-like*	140	n.d.	n.d.
α-Pinene	€	*Terpene-like*	6	n.d.	n.d.
Ethyl 2-methyl butanoate	€	*Fruity*	1	n.d.	n.d.
3-Isobutyl-2-methoxypyrazine		*Bell pepper-like*	10	n.d.	n.d.
(E)-β-Damascenone	€	*Apple boiled-like*	0.002	n.d.	n.d.
(E,E)-2,4-Nonadienal	€	*Fatty*	0.09	n.d.	n.d.
1-Octen-3-one		*Mushroom-like*	0.005	n.d.	n.d.
3-Methylbutyric acid	€	*Sweaty*	120–700	n.d.	n.d.
2-Methylbutyric acid	€	*Sweaty*	\	n.d.	n.d.
Nonanal		*Soapy, Fatty*	1 (in water)	n.d.	n.d.
2-Phenylethanol		*Honey-like*	750–1,110	n.d.	n.d.
(E)-2-Nonenal	€	*Fatty*	0.08–1	n.d.	n.d.
(Z)-2-Decenal	€	*Fatty*	0.3–0.4	n.d.	n.d.
4-Methylphenol		*Horse-like*	55	n.d.	n.d.
(E)-2-Decenal	€	*Fatty*	0.3–0.4	n.d.	n.d.
2-Methoxy-3-sec-butylpyrazine	€	*Bell pepper-like*	0.001 (in water)	n.d.	n.d.
3,5-Dimethyl-2-methoxypyrazine		*Bell pepper-like*	\	n.d.	n.d.
Linalool	€	*Flowery*	6	n.d.	n.d.
Limonene	€	*Lemon-like*	10	n.d.	n.d.
Butyric acid		*Sweaty*	240	n.d.	n.d.
Pentanal		*Moldy*	12–42	n.d.	n.d.
Phenylacetaldehyde		*Honey-like*	4	n.d.	n.d.
4-Ethenyl-2-methoxyphenol (Guaiacol)		*Spicy, phenolic*	3–21	n.d.	n.d.
γ-Octalactone	€	*Fruity coconut-like*	400	n.d.	n.d.
Hexanoic acid		*Sweaty*	3,000	n.d.	n.d.
3,6-Dimethyl-2-ethylpyrazine		*Earthy*	0.4	n.d.	n.d.
4-Hydroxy-3-methoxybenzaldehyde (Vanillin)		*Earthy*	20–200	n.d.	n.d.
€) Molecules presenting stereoisomers					

*Key-aroma compounds (OAV > 1) are reported in italic. n.d.: not determined.*

### Raw Hazelnuts—Cultivar and Geographical Origin Volatiles Signature

The targeted profiling evaluates the response data from selected *m/z* for each compound (Supplementary Table 1); it was applied on all raw hazelnuts’ analyses. The data matrix dimensioned 42 × 91 consisted of mean response values from replicated runs [(14 batches × 3 aliquots)] × 91 targeted compounds. Data were explored through unsupervised and supervised statistics. Averaged responses corresponding to the 14 batches analyzed are provided in Supplementary Table 5.

**FIGURE 1:**
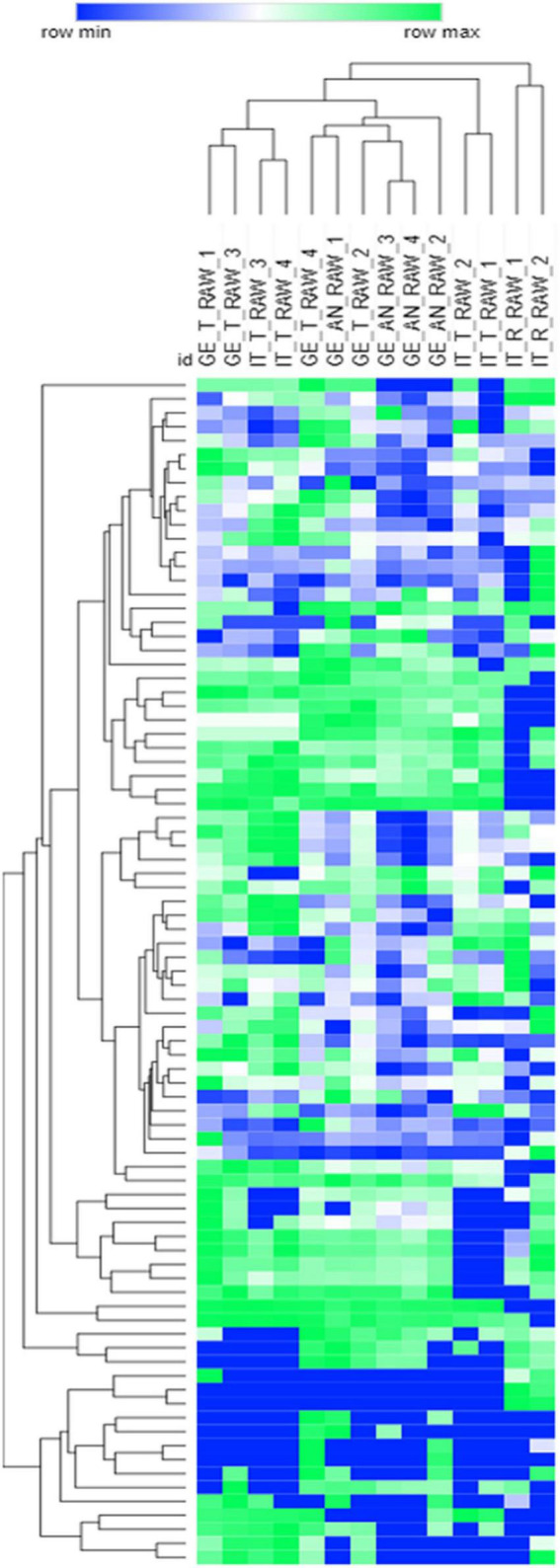
Hierarchical clustering based on Euclidean distances of the raw *Tonda Gentile Trilobata*, *Tonda Gentile Romana*, and *Anakliuri* samples harvested in Italy and Georgia. The response data obtained by averaging batch replicates were log2 normalized; heat-map visualization is in a color scale from blue to green.

The unsupervised investigation by HC, based on Pearson correlation and the response data normalized by Z-score, was performed to reveal the natural groupings existing between samples. Heat-map visualization of HC results is reported in Figure 1 (to note, to simplify the visualization, samples’ response data are reported as mean values). In particular, the interest was on the influence of cultivar and/or geographical origin in delineating diagnostic/discriminant signatures. The HC results show a natural clustering of samples mainly influenced by the cultivar, with the harvesting region being a confounding variable (i.e., some *Tonda Gentile Trilobata* from Georgia clustered together with Georgian *Anakliuri*).

**FIGURE 2:**
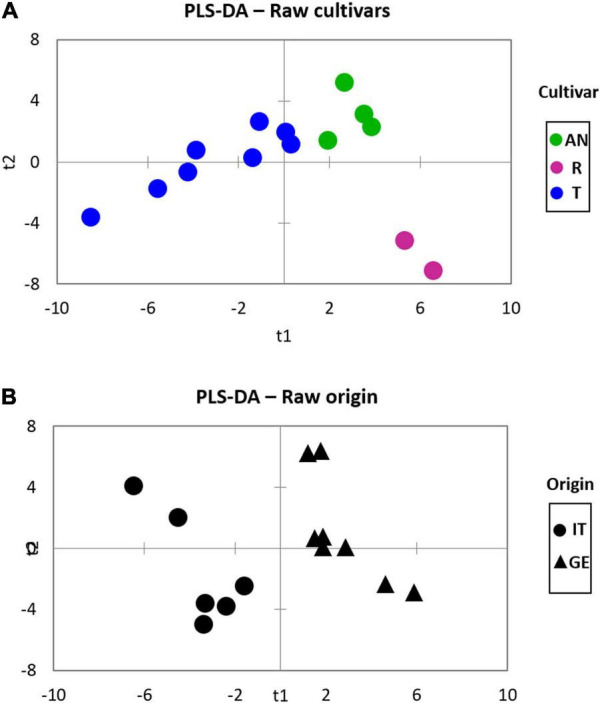
The partial least square discriminant analysis (PLS-DA) of *Tonda Gentile Trilobata*, *Tonda Gentile Romana*, and *Anakliuri* samples harvested in Italy and Georgia focusing on **(A)** cultivar and **(B)** origin on raw samples.

Supervised statistics, in the form of partial least square discriminant analysis (PLS-DA), was then adopted to highlight variables with a higher informative role in describing genetic differences or pedoclimatic conditions on volatiles chiral signatures. Figure 2 shows the plots resulting from the application of the PLS-DA model to the classification of hazelnuts belonging to different cultivars (Figure 2A) while in Figure 2B the model refers to harvest regions.

About cultivars, PLS-DA modeling is coherent with the HC results shown in Figure 1, highlighting that cultivar is the major functional variable impacting on volatiles signature. About origin, Figure 2B shows that within volatiles, some informative markers help in discriminating hazelnuts harvested in Italy from those from Georgia. This discrimination is even more pronounced for the *Tonda Gentile Trilobata;* samples harvested in their native area (T-Italy) have a different fingerprint of those implanted in Georgia (T-Georgia) (Supplementary Figure 2A). This suggests that the phenotype expression of targeted volatiles (see below) is also influenced by pedoclimatic conditions. Figures of merit for the PLS-DA model provided the best performances in terms of accuracy (100%), sensitivity (100%), and specificity (100%) for all the considered classifications.

Then, Variable Importance in Projection (VIP_S_) was used as a feature selection strategy to recognize the predictors that mostly discriminate cultivars and origins: compounds having VIP value ± *SD* higher than 1 were considered significant (Supplementary Figure 3). The list of analytes discriminating the different cultivar includes both chiral (γ-pentalactone, γ-heptalactone, 3-hydroxy-2-butanone, α-pinene) and non-chiral compounds (2-pentanone, 2-heptanone, 2-undecanone, ethanol, acetic acid, acetaldeide, decanal, benzaldehyde, and octanenitrile). Among these, (*R*)-γ-heptalactone (characterized by *creamy*, *coconut*, and *woody* notes; see Supplementary Table 4) was detected in a higher amount in the *Tonda Gentile Romana* (R) cultivar compared to the *Tonda Gentile Trilobata* (T) and *Anakliuri* (AN) cultivars. Similar behavior was noticed for (S)-α-pinene that shows *harsh*, *terpene*-*like*, *coniferous* notes; for (R)-γ-pentalactone; and for the two enantiomers of 3-hydroxy-2-butanone (*buttery* notes). 2-Pentanone, decanal, octanenitrile, and ethyl octanoate were below the method’s limit of detection (LoD) in raw R samples.

About geographical origin discrimination, the list of compounds having a VIP value ± *SD* higher than 1 included both enantiomers of filbertone, 2-heptanone, 2-nonanone, sabinene, (*E*)-2-hexenal, (*E*)-2-octenal, and (*E*)-2-nonenal. Despite the lower number of discriminating compounds, better differentiation between the two groups can be noticed with a higher amount of ketones (filbertone, 2-heptanone, 2-nonanone) in the samples harvested in Italy and an opposite behavior for the aldehydes [(*E*)-2-hexenal, (*E*)-2-octenal and (*E*)-2-nonenal]. The latter group is informative of rancidity status (Cialiè Rosso et al., 2018).

### Roasted Hazelnuts—Technological Evolution of the Volatiles Chemical Fingerprinting

It is well known that the roasting process impacts the primary and specialized metabolites of hazelnuts (Kiefl, 2013; Kiefl et al., 2013; Göncüoğlu Taş and Gökmen, 2017; Cialiè Rosso et al., 2018, 2020; Taş and Gökmen, 2019; Rosso et al., 2021). Thermal reactions, triggered by dry-roasting, generate both non-volatile products (melanoidins, deoxyosones, and reductones) and volatiles, including carbonyl derivatives, alcohols, furanones, pyranones, pyrazines, etc. (Belitz et al., 2009).

Lab-scale roasting, carried out on a traditional ventilated oven, was designed to develop a desirable sensory profile matching industrial standards, due to the development of optimal color, texture, and flavor, confirmed by the strong negative correlation observed between key-odorants and precursors distribution under roasting conditions (Cialiè Rosso et al., 2018, 2020; Rosso et al., 2021). Targeted volatiles profiling was therefore applied on all raw and roasted hazelnuts’ samples, with a consequent increase of the number of samples accounted and the compounds monitored, resulting in an 84 × 150 data matrix [(14 batches × 3 aliquots) × 2 processing steps × 150 targeted volatiles].

**FIGURE 3:**
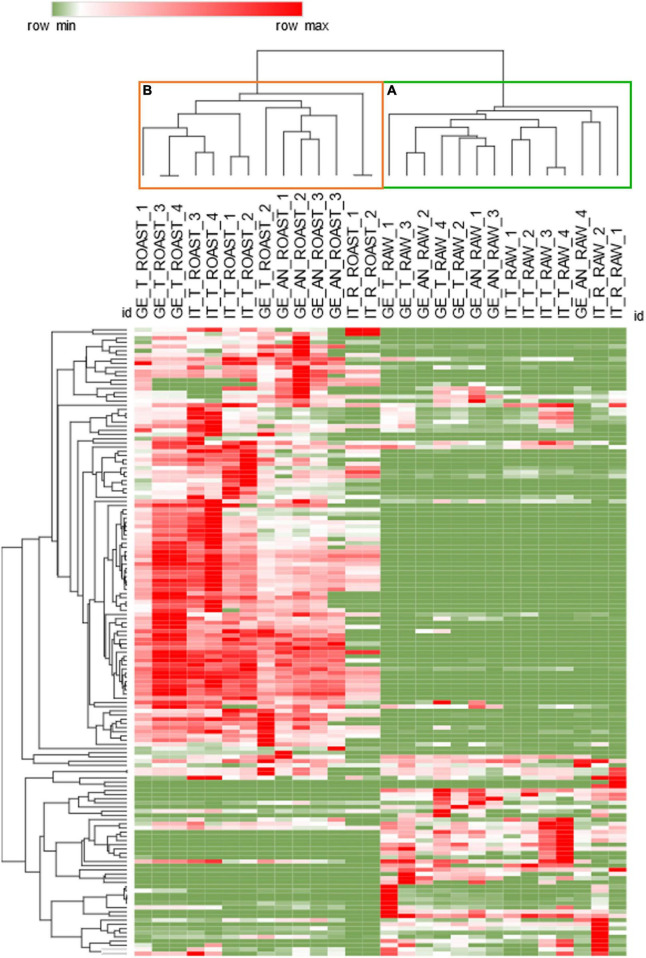
Hierarchical clustering based Pearson’s correlation for raw **(B)** and roasted **(A)** samples. Response data obtained by averaging batch replicates were *Z-score* normalized; heat-map visualization is in a color scale from green to red.

Unsupervised analysis by HC was performed to reveal the natural groupings existing between the samples, characterized by common trends of measured chemical variables. Figure 3 shows the results by the heat-map visualization and the HC based on Pearson correlation after the Z-score normalization of raw response data. The results highlight the major impact of dry-roasting on the volatiles signatures. Cluster *a*, in green, includes all raw hazelnuts, while cluster *b*, in orange, roasted samples. The different height of the branches composing the two clusters indicates that the dry-roasting process increases the variability within the hazelnuts set.

To better investigate the variability between the roasted samples and to understand how roasting differently impacts the natural volatile signature, a further PLS-DA model was developed on a 42 × 117 data matrix [(14 batches × 3 aliquots) × 150 targeted volatiles].

The results are reported in Supplementary Figure 4 with Supplementary Figure 4A showing the score plot resulting from the application of the PLS-DA model on different cultivars, and Supplementary Figure 4B investigating the impact of harvest regions. Results on the cultivars show slightly better discrimination than that obtained on raw samples, showing that the roasting process has a major role in delineating distinctive volatiles signature with a fairly impact on the resulting aroma perception. The impact of roasting has a foundation on the diverse primary metabolites fingerprints and aroma precursors distribution among cultivars (Cialiè Rosso et al., 2018, 2020; Rosso et al., 2021).

**FIGURE 4:**
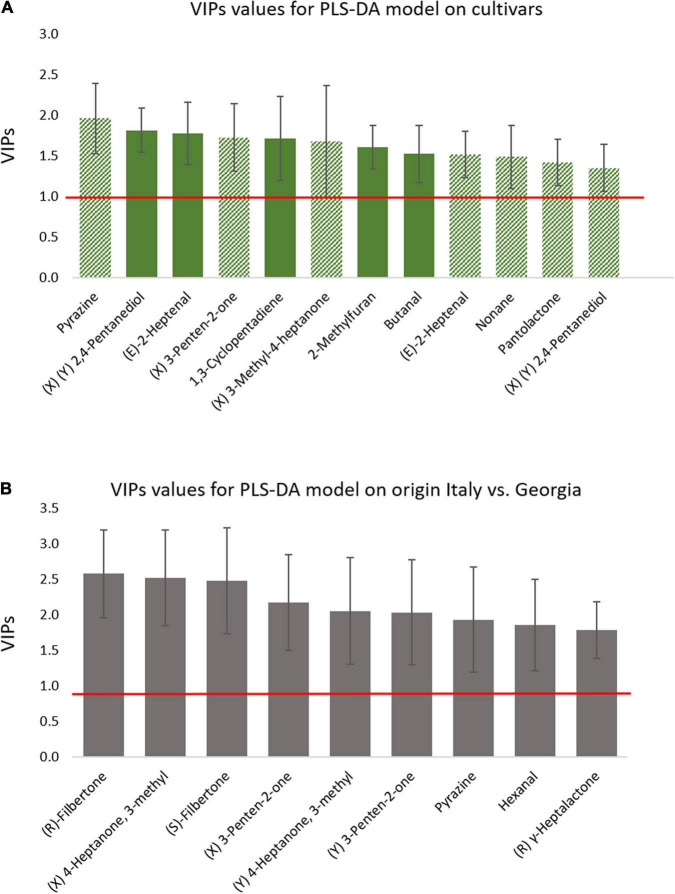
Variable importance in projection (VIP) scores deriving from the PLS-DA based on classification model for roasted samples discriminated for cultivars **(A,B)** origin. Only VIP ± SD > 1 are reported. Bar coloring in **(A)** relates to variables discriminating along the first component (t1-plain color) or along the second (t2—shaded color).

The main compounds responsible for this discrimination (VIP value ± standard deviation higher than 1) include the chiral 3-penten-2-one, 3-methyl-4-heptanone, 2,4-pentanediol, pantolactone, and the not chiral (*E*)-2-heptenal, pyrazine, 1,3-cyclopentadiene, 2-methylfuran, and butanal (Figure 4A). Within these compounds, of particular relevance is 3-methyl-4-heptanone, more abundant in T and AN cultivars than in R samples, it is characterized by *fruity* and *nutty* notes, and was considered a key-odorant for roasted hazelnuts (Kiefl et al., 2013). At the same time, (*E*)-2-heptenal (*fatty, almond*) is higher in AN samples followed by R and T, while 2-methylfuran and butanal discriminate T samples from the other two cultivars for higher absolute amounts.

The PLS-DA model, applied to the classification of hazelnuts harvested in different regions (Supplementary Figure 4B), again provides proofs of the impact of pedoclimatic conditions on phenotype expression including also primary metabolites (Supplementary Figure 2B; Alasalvar et al., 2003b; Cialiè Rosso et al., 2020). In this case, the compounds showing a higher informative role include the two enantiomers of filbertone together with those of 3-methyl-4-heptanone and 3-penten-2-one, (*R*)-γ-heptalactone, and the achiral pyrazine and hexanal (Figure 4B). Again, 3-methyl-4-heptanone, together with filbertone, have a role in the discrimination and, being these compounds key-odorants in the roasted hazelnut, this difference can have a strong impact on the aroma perception of roasted products (Kiefl et al., 2013). Indeed, the two enantiomers of both compounds show higher abundance in Italian samples, thus suggesting more intense *fruity* and *nutty* notes in these samples. In particular, the behavior of filbertone is in agreement with the results reported by Nicolotti et al. (2013a) who observed a strong cultivar/origin-related increment of 5-methyl-(*E*)-2-hepten-4-one with Italian *Tonda Gentile Trilobata* with an early increase of this potent odorant at mild roasting conditions. Conversely, the Georgian samples show a higher abundance of hexanal, which is characterized by *fatty* and *green-leafy* notes (Pastorelli et al., 2007; Ghirardello et al., 2016) thus eliciting possible off-flavors.

### Chiral Signature and Filbertone Enantiomers Distribution

As previously mentioned, chiral components are a relevant part not only of the compounds having an important impact on the hazelnut aroma but also of those showing abundance differences depending on cultivar and pedoclimatic factors and in the comparison between raw and roasted hazelnuts. Moreover, the roasting process might not influence the enantiomeric ratio of some chiral compounds, although the non-stereospecific thermal treatment could differently impact the enantiomeric ratio. In fact, the native enantiomer ratio could change because the probability to form *(R)* or *(S)* forms is univariate unless other enzymatic or additional factors take part in the process. In this perspective, this paragraph deals with the evaluation of the enantiomeric signature of the main hazelnut chiral compounds focusing on the changes and evolution of the enantiomeric ratio in samples after roasting. To note, within the sample set, shelf-life was not examined although represented. However, its influence might not be negligible as suggested by the enantiomeric composition (EC) ranges (see Table 2) that show larger differences for some chiral markers. A particular emphasis will be devoted to the distribution of filbertone enantiomers because of the relevance of this compound not only in determining the hazelnut raw and roasted aroma quality (Kiefl and Schieberle, 2013; Kiefl et al., 2013) but also because its amount is strongly related to the harvesting origin.

**TABLE 2:** Percentage enantiomeric composition of the main chiral markers of the hazelnut volatilome.

	Roasted samples				Raw samples			
Compounds	GE-T	IT-T	GE_AN	ITR	GE-T	IT-T	GE-AN	IT-R
**Terpenes**								
(*R*) Phellandrene	ND	ND	ND	ND	91–99	ND	>99	65–67
(*S*) Phellandrene	ND	ND	ND	ND	1–9	ND	>1	33–35
(*R*) α-Pinene	70–72	48–70	66–78	57–68	71–82	54–73	70–87	55–59
(*S*) α-Pinene	28–30	30–52	22–34	32–43	18–29	27–46	13–30	41–45
(*R*) Limonene	>1	14–19	18–39	ND	30–55	14–19	23–37	5–12
(*S*) Limonene	>99	81–86	61–82	ND	45–70	81–86	63–77	88–95
(*R)* Linalool	69–77	46–58	69–81	ND	65–88	78–82	77–83	ND
(*S)* Linalool	23–31	42–54	19–31	ND	12–35	18–22	17–23	ND
**Lactones**								
(*R*) γ-Pentalactone	20–22	29–31	14–20	20–38	37–49	48–55	37–50	51–47
(*S*) γ-Pentalactone	78–80	69–71	80–86	62–80	51–63	45–52	50–63	49–53
(*R*) γ-Hexalactone	ND	ND	ND	ND	37–50	20–50	45–81	48–49
(*S*) γ-Hexalactone	ND	ND	ND	ND	50–63	80–50	19–65	51–52
(*R*) γ-Octalactone	ND	ND	ND	ND	44–58	30–52	47–50	39–44
(*S*) γ-Octalactone	ND	ND	ND	ND	42–56	48–70	50–53	56–61
(*R*) β-Angelica lactone	48–52	50–55	49–54	NM	ND	ND	ND	ND
(*S*) β-Angelica lactone	48–52	45–50	46–51	NM	ND	ND	ND	ND
**Ketones**								
(*R*)-Filbertone	13–16	27–33	8–11	25–26	8–11	15–17	5–8	10–11
(*S*)-Filbertone	84–81	67–73	89–92	74–75	89–92	83–85	92–95	89–90
(X) 4-Heptanone, 3-methyl	31–48	23–57	34–39	NM	ND	ND	ND	ND
(Y) 4-Heptanone, 3-methyl	52–69	43–77	61–66	NM	ND	ND	ND	ND
(X) 2-Butanone, 3-hydroxy-	48–49	47-48	49–50	48–52	41–42	31–43	35–42	15–27
(Y) 2-Butanone, 3-hydroxy-	51–52	52–53	50–51	48–52	58–59	57–69	57–65	63–85
(X) 4H-Pyran-4-one, 2,3-dihydro-3,5-dihydroxy-6-methyl-	6–11	8–12	6–10	NM	>1	>1	>1	>1
(Y) 4H-Pyran-4-one, 2,3-dihydro-3,5-dihydroxy-6-methyl-	89–94	88–92	90–94	NM	>99	>99	>99	>99
**Alcohols and esters**								
(X) 2-Pentanol	>99	>99	>99	>99	49–47	42–54	NM	48–50
(Y) 2-Pentanol	>1	>1	>1	>1	51–53	46–58	NM	50–52
(*R)* ethyl-2-methylbutanoate	ND	ND	ND	ND	56–73	82–84	ND	ND
(*S*) ethyl-2-methylbutanoate	ND	ND	ND	ND	27–44	16–18	ND	ND
(X) (X) 2,4-Pentanediol	82–86	52–69	83–93	ND	ND	ND	ND	ND
(X) (Y) 2,4-Pentanediol	14–18	31–48	7–17	ND	ND	ND	ND	ND

*ND, not detected; NM, not possible to measure because of interfering compounds. When > 99/ < 1 composition is reported only one enantiomer has been detected.*

Table 2 reports the enantiomeric distribution of the main markers of the hazelnut volatilome in the investigated samples expressed in terms of the range of percentage enantiomeric composition:

$$E\,C\%=\frac{E_{1}}{E_{1}+E_{2}}\times 100$$

where *E*_1_ and *E*_2_ are the chromatographic areas (i.e., absolute responses) of the two enantiomers.

The enantiomeric distribution of native terpenes represents a natural signature of the plant since their biosynthetic pathway follows an enzymatic pathway from C_5_ precursors. Their biosynthesis is therefore often stereo-guided and results in an enantiomeric excess of one of the two enantiomers. In addition, as reported in Supplementary Table 4, the relative abundance of the two enantiomers can impact the overall aroma perception of hazelnut since the odor quality of the two enantiomers can substantially differ. For α-pinene and limonene, a higher abundance of the *(S)* enantiomer can be noted in all the investigated samples with no substantial differences between raw and roasted nuts. α-Phellandrene was detected only in raw samples (apart IT-T) always with a strong enantiomeric excess for the (*R*) enantiomer. Particular attention should be paid to the enantiomeric composition of linalool since it is considered one of the most impacting odorants of raw hazelnuts (Burdack-Freitag and Schieberle, 2010, 2012), with the two enantiomers eliciting different notes [i.e., *floral*, *woody lavender* for the (*R*)-linalool and *sweet*, *floral* for the (*S*) enantiomer]. Linalool was detected in raw and roasted samples, with the exception of the *Tonda Gentile Romana* available for this study. Due to the critical resolution achieved for linalool enantiomers, the enantiomeric composition should be confirmed by adopting a different GC chiral selector. However, an excess of the (*R*)-enantiomer is reasonably correlated to the intense *floral* perception in the raw hazelnut aroma accompanied by *woody* notes (Burdack-Freitag and Schieberle, 2010, 2012).

Ethyl 2-methylbutanoate is also reported as a key-aroma compound in raw hazelnuts; it was detected in *Tonda Gentile Trilobata* samples with a higher abundance for the (*R*)-enantiomer that is characterized by a *fruity-sweet* note as reported also by Burdack-Freitag and Schieberle (2010, 2012).

Lactones are other important chiral markers of the hazelnut volatilome and also show different odor attributes depending on their configuration. Biosynthesis can follow a chemical or enzymatic pathway (Romero-Guido et al., 2011). The distribution of lactones within the investigated raw samples varies in a quite wide range from almost racemic to high abundance of one enantiomer (in particular for γ-hexalactone). As it is known, lactones can be enzymatically formed after yeast and/or fungi attack (Romero-Guido et al., 2011), it would be therefore interesting to further evaluate if the change in the enantiomeric composition should be correlated to spoilage (Stilo et al., 2021). In addition, it should be noted that in most cases, the lactones cannot be detected in roasted samples; however, γ-pentalactone shows an increase of the (*S*)- enantiomer and angelica lactone (i.e., 5-methyl-2 (3H)-furanone) can be detected only in roasted samples; its racemic distribution allows to hypothesize a chemical pathway of formation during the thermal treatment.

Among the other chiral compounds (chiefly alcohols and ketones), particular attention should be focused on filbertone and 3-methyl-4-heptanone since they are key-aroma compounds and, as highlighted in the previous paragraph, their abundance considerably changes according to the cultivar and/or origin. 3-Methyl-4-heptanone is a key odorant in roasted hazelnut and shows an enantiomeric excess of one of the two enantiomers. However, it was not possible to assign the configuration because of the lack of pure enantiomeric standards. In addition, the odor properties of the single isomers have not yet been investigated, meaning that it is not possible to establish if the perception of this compound can be related to its enantiomeric distribution. Further investigations are underway in this respect.

**FIGURE 5:**
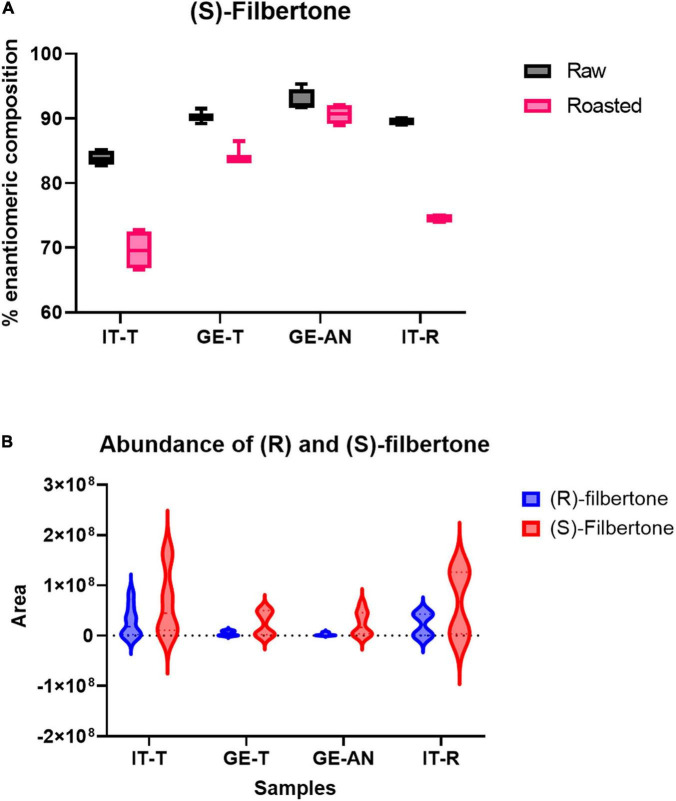
**(A)** Percentage enantiomeric composition of (*S*)-Filbertone in raw and roasted hazelnut from *Tonda Gentile Trilobat*a harvested in Italy and Georgia (IT-T and GE-T), *Tonda Gentile Romana* harvested in Italy (IT-R), and *Anakliuri* harvested in Georgia (GE-AN). **(B)** Violin plot of raw areas of (*R*) and (*S*) Filbertone (blue and red, respectively) in raw and roasted samples.

Figure 5A reports the box-plot visualization of the percentage enantiomeric composition of (*S*)-filbertone for the investigated samples. The graphs show that (*S*)-filbertone is the more abundant isomer in all samples with quite high and uniform enantiomeric excess in raw hazelnuts (percentage enantiomeric composition between 83 and 95%). The relative difference between the two enantiomers drops down to lower enantiomeric excess after roasting and in all samples, as already reported in the literature (Güntert et al., 1991; Blanch and Jauch, 1998; Puchl’ová and Szolcsányi, 2018), with a higher difference for Italian samples. To better investigate the relative amount of the two enantiomers in the investigated cultivars, *violin* plots reporting the distribution of their absolute abundances were created (Figure 5B). The graphs show a bimodal distribution of the abundance of both enantiomers, higher values are observed in all cases after roasting. This result is in keeping with the reference research by Pfnuer et al. (1999). Interestingly the increment is more pronounced for the (*R*)-enantiomer than the (*S*)-enantiomer. It can be also noticed that, as already observed, the hazelnuts harvested in Italy show higher amounts of both filbertone enantiomers compared to the Georgian samples. This difference is particularly marked for (*R*)-filbertone and results in the lower enantiomeric excess (see above). This distribution impacts on the aroma perception since it is known that the (*-*)-(*R*)-(*E*)- 5-methyl-2-hepten-4-one has more pleasant *hazelnut* and *woody* odor notes and a *soft, butter, chocolate* flavor impact (Güntert et al., 1991). Its higher abundance in the Italian samples (in particular for the *Tonda Gentile Trilobata*) supports its characteristic *nutty-fruity* notes (Kiefl and Schieberle, 2013) and the higher perceived appreciation by the consumers that makes this cultivar the highest quality standard for confectionery industries.

## Conclusion

The present study evaluated, for the first time, the hazelnut volatilome with the perspective of providing insights into the enantiomeric distribution of native and newly formed chiral components. The attention was focused in particular on the influence of functional variables, such as cultivar and pedoclimatic conditions, and the technological impact exerted by the roasting process. The results showed that chiral volatile patterns of hazelnuts belonging to different cultivars and harvested in different regions compounds have distinctive signatures. Moreover, being chiral molecules often key-aroma compounds, their distribution can have a strong impact on the sensory properties since the enantiomers of the same molecules can substantially differ in terms of odor descriptors and/or threshold.

In this sense, the chiral recognition of the two enantiomers of the key-aroma (*E*)-5-methyl-2-hepten-4-one (filbertone) provides fundamental insights for cultivar and geographical authentication, while suggesting differential organoleptic properties. The results showed that the synthesis of the (*R*)- and (*S*)-filbertone is affected not only by genetic (cultivar) factors but also by environmental pressure. As a consequence, the higher amount of the (*R*)-enantiomer for hazelnuts harvested in Italy results in a more appreciated *nutty* aroma if compared with other samples.

To disclose the real potential of the chiral volatilome, further studies are required to extend the evaluation to a wider range of cultivars and origins and to deepen the impact of shelf-life/storage. Moreover, the odor properties and the distribution of chiral markers (such as 3-methyl-4-heptanone), not yet evaluated in terms of single enantiomers, deserve a more in-depth investigation that would be helpful to correlate the hazelnuts chemical fingerprint to the hedonic profile. In an *omic* approach, the enantiomeric recognition of a plant/food volatilome increases the level of information to a further step enabling, at the same time, to better disclose the influence of functional variables on the matrix chemical pattern and metabolic pathway, and to better relate it to the sensory perception.

## Data Availability Statement

The original contributions presented in the study are included in the article/Supplementary Material, further inquiries can be directed to the corresponding author/s.

## Author Contributions

FS: investigation, writing—original draft, writing—review and editing. MC: formal analysis, data curation, writing—review and editing. SS: data curation, visualization, and writing—review and editing. CB: resources, writing—review and editing, and supervision. CCo: conceptualization, methodology, visualization, writing—original draft, writing—review and editing, project administration, and supervision. CCa: conceptualization, methodology, data curation, writing—original draft, and writing—review and editing. All authors contributed to the article and approved the submitted version.

## Conflict of Interest

FS was employed by the Laemmegroup S.r.l - A Tentamus Company. The remaining authors declare that the research was conducted in the absence of any commercial or financial relationships that could be construed as a potential conflict of interest.

## Publisher’s Note

All claims expressed in this article are solely those of the authors and do not necessarily represent those of their affiliated organizations, or those of the publisher, the editors and the reviewers. Any product that may be evaluated in this article, or claim that may be made by its manufacturer, is not guaranteed or endorsed by the publisher.

## Footnotes

1
https://software.broadinstitute.org/GENE-E/
