# Supplementary material for: Corylus avellana L. Natural Signature: Chiral Recognition of Selected Informative Components in the Volatilome of High-Quality Hazelnuts
Source: Front Plant Sci. 2022 Apr 25;13:844711. doi: 10.3389/fpls.2022.844711 (PMC9085359; doi:10.3389/fpls.2022.844711)
Supplement: Supplementary file 1 [file Data_Sheet_1.zip › Supplementary material.docx]

*SUPPLEMENTARY MATERIAL*

*Corylus avellana* L. natural signature: chiral recognition of selected informative components in the volatilome of high-quality hazelnuts

Federico Stilo^1,2^, Marta Cialiè Rosso^1^, Simone Squara^1^, Carlo Bicchi^1^, Chiara Cordero^1^*, Cecilia Cagliero^1^

^1^ Dipartimento di Scienza e Tecnologia del Farmaco, Università degli Studi di Torino, Turin, Italy

^2^ Laemmegroup - A Tentamus Company, Moncalieri, Torino, Italy

* Correspondence:

Corresponding Author

chiara.cordero@unito.it

**Tables**

**Supplementary Table S1.** Targeted compounds detected in raw and roasted hazelnut samples with the experimental *I^T^* on MeAc-TBDM-β-CD column. # represents chiral compounds not separated by the chiral selector, § chiral compounds whose elution order of (*R*) and (*S*) enantiomers was known, * chiral compounds whose enantiomer elution order is not yet known (X indicates the first eluted, while Y the last eluted) and ^ chiral compound for which only one enantiomer was found. Analytical method precision on response data from replicated analyses performed over two-weeks on roasted *Tonda Gentile Romana*.

| **IUPAC names** | **Trivial names** | **Notation** | **Exp. *I^T^*** | ***Ti* (m/z)** | **Detected in Raw** | **Detected in Roasted** | **Precision**  **(RSD%)** |
| --- | --- | --- | --- | --- | --- | --- | --- |
| (1R,2R,5S)-5-Isopropyl-2-methylbicyclo | (S)-(-)-Z-Sabinene hydrate | § | 1337 | 71 | X | X | 0.70 |
| (1R,5R)-2,6,6-Trimethylbicyclo[3.1.1]hept-2-ene | (R)-(+)-α-Pinene | § | 943 | 93 | X | X | 13.73 |
| (1S,2S,5R)-5-Isopropyl-2-methylbicyclo-[3.1.0]-hexan-2-ol | (R)-(+)-(Z)-Sabinene hydrate | § | 1326 | 71 | X |  |  |
| (1S,5S)-2,6,6-Trimethylbicyclo[3.1.1]hept-2-ene | (S)-(-)-α-Pinene | § | 937 | 93 | X | X | 0.32 |
| (2E,5R)-5-Methyl-2-hepten-4-one | (R)-Filbertone | § | 1173 | 98 | X | X | 3.23 |
| (2E,5S)-5-Methyl-2-hepten-4-one | (S)-Filbertone | § | 1210 | 98 | X | X | 3.33 |
| (2Z,5R)-5-Methyl-2-hepten-4-one |  | § | 1044 | 69 | X |  |  |
| (2Z,5S)-5-Methyl-2-hepten-4-one |  |  | 1050 | 69 | X |  |  |
| (3R)-3,7-Dimethylocta-1,6-dien-3-ol | (R)-Linalool | § | 1390 | 71 | X | X | 11.29 |
| (3S)-3,7-Dimethylocta-1,6-dien-3-ol | (S)-Linalool | § | 1392 | 71 | X | X | 1.61 |
| (4R)-4-Isopropenyl-1-methylcyclohexene | (R)-Limonene | § | 1059 | 68 | X | X | 5.61 |
| (4S)-4-Isopropenyl-1-methylcyclohexene | (S)-Limonene | § | 1063 | 68 | X | X | 0.74 |
| (5R)-2-Methyl-5-propan-2-ylcyclohexa-1,3-diene | (R)-(-)-α-Phellandrene | § | 1042 | 93 | X |  |  |
| (5S)-2-Methyl-5-propan-2-ylcyclohexa-1,3-diene | (S)-(+)- α-Phellandrene | § | 1046 | 93 | X |  |  |
| (E)-2-Butenal |  |  | 1094 | 70 |  | X | 3.06 |
| (E)-2-Decenal |  | € | 1626 | 70 | X | X | 1.38 |
| (E)-2-Heptenal |  |  | 1367 | 83 | X | X | 0.80 |
| (E)-2-Hexenal |  |  | 1263 | 83 | X |  |  |
| (E)-2-Methyl-2-butenal |  |  | 1215 | 55 |  | X | 11.55 |
| (E)-2-Nonenal |  | € | 1543 | 70 | X |  |  |
| (E)-2-Octenal |  |  | 1456 | 70 | X | X | 0.36 |
| (E)-2-Undecenal |  |  | 1712 | 83 | X | X | 11.49 |
| (E,E)-2,4-Decadienal |  | € | 1701 | 81 |  | X | 6.92 |
| (E,E)-2,4-Nonadienal |  | € | 1615 | 81 | X |  |  |
| (R)-5-Butyldihydro-2(3H)-furanone | (R)-γ-Octalactone | § | 1849 | 85 | X |  |  |
| (R)-5-Ethyldihydro-2(3H)-furanone | (R)-γ-Hexalactone | § | 1742 | 85 | X |  |  |
| (R)-5-Methyl-2(5H)-furanone | (R)-β-angelica lactone | § | 1787 | 55 |  | X | 0.92 |
| (R)-Dihydro-5-methyl-2(3H)-furanone | (R)-γ-Pentalactone | § | 1751 | 56 | X | X | 15.33 |
| (R)-Dihydro-5-pentyl-2(3H)-furanone | (R)-γ-Nonalactone | § | 1941 | 85 | X | X | 1.00 |
| (R)-Dihydro-5-propyl- 2(3H)-furanone | (R)-γ-Heptalactone | ^§ | 1774 | 85 | X |  |  |
| (R)-Ethyl-2-methylbutanoate | (R)-Ethyl-2-methylbutyrate | § | 928 | 102 | X | X | 8.79 |
| (S)-5-Butyldihydro-2(3H)-furanone | (S)-γ-Octalactone | § | 1882 | 85 | X |  |  |
| (S)-5-Ethyldihydro- 2(3H)-furanone | (S)-γ-Hexalactone | § | 1780 | 85 | X |  |  |

| **IUPAC names** | **Trivial names** | **Notation** | **Exp. *I^T^*** | ***Ti* (m/z)** | **Detected in Raw** | **Detected in Roasted** | **Precision**  **(RSD%)** |
| --- | --- | --- | --- | --- | --- | --- | --- |
| (S)-5-Methyl-2(5H)-furanone | (S)-β-angelica lactone | § | 1865 | 55 |  | X | 7.02 |
| (S)-Dihydro-5-methyl-2(3H)-furanone | (S)-γ-Pentalactone | § | 1791 | 56 | X | X | 6.14 |
| (S)-Dihydro-5-pentyl-2(3H)-furanone | (S)-γ -Nonalactone | § | 1971 | 85 | X | X | 4.31 |
| (S)-Ethyl-2-methylbutanoate | (S)-Ethyl-2-methylbutyrate | § | 940 | 102 | X | X | 4.21 |
| (X) (X)-2,3-Butanediol |  | * | 1455 | 45 | X | X | 5.98 |
| (X) (X)-2,4-Pentanediol |  | * | 1531 | 45 |  | X | 1.58 |
| (X) (Y)-2,3-Butanediol |  | * | 1478 | 45 | X | X | 2.42 |
| (X) (Y)-2,4-Pentanediol |  | * | 1527 | 45 |  | X | 2.01 |
| (X)-2,3-Dihydro-3,5-dihydroxy-6-methyl-4H-pyran-4-one |  | * | 1730 | 144 |  | X | 4.16 |
| (X)-2-Pentanol |  | * | 1024 | 45 | X |  |  |
| (X)-3-Hydroxy-2-butanone |  | * | 1376 | 88 | X | X | 11.53 |
| (X)-3-Methyl-4-Heptanone |  | * | 1042 | 57 |  | X | 0.13 |
| (X)-3-Penten-2-one |  | * | 1215 | 84 | X | X | 11.64 |
| (Y) (X)-2,3-Butanediol |  | * | 1433 | 45 | X | X | 0.71 |
| (Y) (X)-2,4-Pentanediol |  | * | 1536 | 45 |  | X | 0.07 |
| (Y)-2,3-Dihydro-3,5-dihydroxy-6-methyl-4H-pyran-4-one |  | * | 1754 | 144 |  | X | 4.27 |
| (Y)-2-Pentanol |  | * | 1027 | 45 | X |  |  |
| (Y)-3-Hydroxy-2-butanone |  | * | 1416 | 88 | X | X | 16.52 |
| (Y)-3-Methyl-4-Heptanone |  | * | 1055 | 57 |  | X | 11.08 |
| (Y)-3-Penten-2-one |  | * | 1216 | 84 | X | X | 3.21 |
| 1,3-Cyclopentadiene |  |  | <900 | 66 | X | X | 3.84 |
| 1-Decene |  |  | 1011 | 70 | X |  |  |
| 1-Ethyl-1H-pyrrole |  |  | 1198 | 80 |  | X | 0.29 |
| 1-Heptanol |  |  | 1230 | 70 | X |  |  |
| 1-Hexanol |  |  | 1146 | 56 | X |  |  |
| 1-Hydroxy-2-propanone |  |  | 1295 | 43 |  | X | 2.88 |
| 1-Isopropyl-4-methylbicyclo[3.1.0]hex-3-ene | α-Thujene | # | 941 | 93 | X |  |  |
| 1-Methyl-2-pyrrolidinone |  |  | 1705 | 99 |  | X | 6.11 |
| 1-Nonene |  |  | 912 | 56 | X |  |  |
| 1-Octanol |  |  | 1316 | 56 | X |  | 1.86 |
| 1-Pentanol |  |  | 1058 | 55 |  | X | 1.25 |
| 2(5H)-Furanone | γ -Crotonolactone |  | 1853 |  |  | X | 10.89 |
| 2,3,5-Trimethylfuran |  |  | <900 | 110 |  | X | 0.46 |
| 2,3-Dimethylpyrazine |  |  | 1210 | 108 |  | X | 2.85 |
| 2,5-Diethylpyrazine |  |  | 1304 | 121 |  | X | 12.48 |
| 2,5-Dimethyl-4-hydroxy-3(2H)-furanone | Furaneol | # | 1638 | 128 |  | X | 9.57 |
| 2,5-Dimethylfuran |  |  | <900 | 96 |  | X | 2.02 |
| 2,5-Dimethylpyrazine |  |  | 1120 | 108 |  | X | 2.02 |

| **IUPAC names** | **Trivial names** | **Notation** | **Exp. *I^T^*** | ***Ti* (m/z)** | **Detected in Raw** | **Detected in Roasted** | **Precision**  **(RSD%)** |
| --- | --- | --- | --- | --- | --- | --- | --- |
| 2,6-Dimethylpyrazine |  |  | 1149 | 108 |  | X | 8.13 |
| 2-Acetylpyridine |  |  | 1407 | 79 |  | X | 3.77 |
| 2-Butanone |  |  | 1026 | 43 |  | X | 4.24 |
| 2-Carboxaldehyde-1H-pyrrole | Formyl pyrrole |  | 1581 | 95 |  | X | 2.40 |
| 2-Cyclopentene-1,4-dione |  |  | 1578 | 96 |  | X | 6.60 |
| 2-Decanone |  |  | 1435 | 58 | X | X | 9.37 |
| 2-Ethyl-5-methylpyrazine |  |  | 1225 | 122 |  | X | 9.11 |
| 2-Ethyl-6-methylpyrazine |  |  | 1200 | 121 |  | X | 14.26 |
| 2-Furancarboxaldehyde | Furfural |  | 1364 | 96 |  | X | 4.86 |
| 2-Furanmethanol |  |  | 1276 | 98 |  | X | 6.95 |
| 1-(2-Furyl)-2-hydroxyethanone | 2-Furylhydroxymethyl ketone |  | 1644 | 95 |  | X | 0.79 |
| 2-Heptanone |  |  | 1165 | 58 | X | X | 1.27 |
| 2-Methyl-1H-pyrrole |  |  | 1229 | 121 |  | X | 3.27 |
| 2-Methylbutanal |  | # | <900 | 41 | X | X | 0.15 |
| 2-Methylfuran |  |  | <900 | 82 |  | X | 1.98 |
| 2-Methylpentane |  |  | <900 | 43 | X | X | 3.36 |
| 2-Methylpropanal |  |  | 991 | 43 |  | X | 8.36 |
| 2-Nonanone |  |  | 1347 | 58 | X |  |  |
| 2-Octanone |  |  | 1259 | 58 | X |  |  |
| 2-Pentanone | Ethyl acetone |  | 1065 | 43 | X | X | 8.51 |
| 2-Phenyl-2-butenal |  |  | 1493 | 117 |  | X | 18.14 |
| 2-Propanone | Acetone |  | 919 | 43 | X | X | 3.38 |
| 2-Pyrrolidinone |  |  | 1958 | 42 |  | X | 6.95 |
| 2-Undecanone |  |  | 1525 | 71 | X | X | 2.66 |
| 2-Vinylfuran |  |  | <900 | 94 |  | X | 14.64 |
| 3,5,5-Trimethyl-2(5H)-furanone |  | € | 1933 |  |  | X | 6.31 |
| 3-Ethyl-2,5-dimethylpyrazine |  |  | 1234 | 135 |  | X | 2.44 |
| 3-Hexanone |  |  | 1026 | 71 |  | X | 2.49 |
| 3-Hydroxy-2-methyl-4H-pyran-4-one |  |  | 1720 | 126 |  | X | 7.79 |
| 3-Methyl-1H-pyrrole |  |  | 1296 | 80 |  | X | 12.89 |
| 3-Methyl-2(5H)-furanone | α-Methyl-γ-crotonolactone |  | 1816 | 98 |  | X | 8.09 |
| 3-Methyl-2-cyclopenten-1-one |  |  | 1630 | 96 |  | X | 3.18 |
| 3-Methyl-2-pentanone |  |  | 1110 | 43 |  | X | 2.69 |
| 3-Methylbutanal |  | € | 1031 | 58 |  | X | 11.24 |
| 3-Methylphenol |  |  | 935 | 108 |  | X | 1.74 |
| 3-Octen-2-one |  |  | 1419 | 111 | X |  |  |
| 3-Penten-2-ol |  |  | 998 | 71 |  | X | 5.64 |
| 3-Undecanone |  |  | 1462 | 72 | X |  |  |
| 4-Heptanone |  |  | 1055 | 71 |  | X | 1.64 |
| 4-Methyl-2(5H)-furanone |  |  | 2075 | 98 |  | X | 0.21 |
| 4-methylene-1-(1-methylethyl) bicyclo[3.1.0]hexane | Sabinene | # | 1098 | 93 | X |  |  |

| **IUPAC names** | **Trivial names** | **Notation** | **Exp. *I^T^*** | ***Ti* (m/z)** | **Detected in Raw** | **Detected in Roasted** | **Precision**  **(RSD%)** |
| --- | --- | --- | --- | --- | --- | --- | --- |
| 4-Octen-3-one |  |  | 1227 | 97 | X |  |  |
| 5,6-Dihydro-2H-pyran-2-one |  |  | 2014 | 98 |  | X | 0.49 |
| 5,6-Dihydro-6-pentyl-2H-pyran-2-one | C-10-Massoia lactone | # | 1905 | 55 |  | X | 2.89 |
| 5-Ethyl-2,3-dimethylpyrazine |  |  | 1293 | 135 |  | X | 16.31 |
| 5-Hydroxymethyl-2-furancarboxaldehyde | 5-(hydroxymethyl)-furfural |  | 1931 | 126 |  | X | 1.04 |
| 6-Methyl-5-hepten-2-one |  |  | 1218 | 108 | X |  |  |
| Benzaldehyde |  |  | 940 | 106 | X | X | 3.48 |
| Butanal |  |  | 977 | 72 |  | X | 11.26 |
| Decanal |  |  | 1448 | 70 | X |  |  |
| Decane |  |  | 995 | 57 | X |  |  |
| Dihydro-2(3H)-furanone | γ -Butyrolactone |  | 1807 | 42 | X |  |  |
| Dihydro-3-hydroxy-4,4-dimethyl-2(3H)-furanone | Pantolactone | # | 1998 | 71 | X | X | 1.47 |
| Dodecane |  |  | 1200 | 57 | X |  |  |
| Ethanal | Acetaldehyde |  | <900 | 44 | X |  |  |
| Ethanoic acid | Acetic acid |  | 1144 | 60 | X | X | 0.14 |
| Ethanol |  |  | <900 | 31 | X |  |  |
| Ethenyl hexanoate | Vinyl caproate |  | 1541 | 71 | X |  |  |
| Ethyl acetate |  |  | 1517 | 43 |  | X | 13.46 |
| Ethyl octanoate |  |  | 1301 | 88 | X |  |  |
| Ethylpyrazine |  |  | 1154 | 107 |  | X |  |
| Heptanal |  |  | 1183 | 70 | X | X | 3.05 |
| Heptane |  |  | <900 | 43 | X | X | 2.52 |
| Hexanal | Caproaldehyde |  | 1083 | 56 | X | X | 1.98 |
| Hexane |  |  | <900 | 57 | X | X | 3.36 |
| Hexanoic acid |  |  | 1432 | 60 | X |  |  |
| Methoxyphenyloxime |  |  | 1397 | 151 |  | X | 8.36 |
| Methyl ethanoate | Methyl acetate |  | <900 | 43 | X | X | 3.44 |
| Methyl nonanoate |  |  | 1340 | 74 | X |  |  |
| Methyl octanoate |  |  | 1246 | 74 | X |  |  |
| Methyl-(1-methylethyl)-benzene | Cymene |  | 1112 | 119 | X |  |  |
| Methyl-2-propenoate |  |  | <900 | 55 |  | X | 8.51 |
| Methyl-2-pyridinecarboxylate |  |  | 1650 | 79 |  | X | 15.14 |
| Methylpyrazine |  |  | 1088 | 94 |  | X | 3.38 |
| Nonanal |  |  | 1356 | 57 | X | X | 6.95 |
| Nonane |  |  | 899 | 57 | X | X | 2.66 |
| Octanal |  |  | 1268 | 84 | X | X | 8.17 |
| Octane |  |  | <900 | 43 | X | X | 0.87 |
| Octanenitrile |  |  | 1536 | 82 | X |  |  |
| Pentanal | Valeraldehyde |  | 1027 | 44 | X | X | 10.38 |
| Phenylformamide |  |  | <900 | 121 |  | X | 0.15 |
| Pyrazinamide |  |  | 1640 | 123 |  | X | 0.05 |

| **IUPAC names** | **Trivial names** | **Notation** | **Exp. *I^T^*** | ***Ti* (m/z)** | **Detected in Raw** | **Detected in Roasted** | **Precision**  **(RSD%)** |
| --- | --- | --- | --- | --- | --- | --- | --- |
| Pyrazine |  |  | 1016 | 80 |  | X | 0.08 |
| Pyrrole |  |  | 1239 | 67 |  | X | 0.66 |
| Tetrahydro-2H-pyran-2-one | δ-Valerolactone |  | 1975 | 100 | X | X | 0.47 |
| (X) Tetrahydro-6-methyl-2H-pyran-2-one | δ-Hexalactone | ^* | 1850 | 70 |  | X | 1.17 |
| (X) Tetrahydro-6-propyl-2H-Pyran-2-one | δ-Octalactone | # | 1842 | 99 | X | X | 5.68 |
| Trimethylpyrazine |  |  | 1235 | 122 |  | X | 0.03 |
| # chiral compounds not separated by chiral selector  § chiral compounds whose elution order of R and S enantiomers was known  * chiral compounds whose enantiomer elution order is not yet known; X indicates the first eluted, while Y the last eluted  ^ chiral compound for which only one enantiomer was found | | | | | | | |

**Supplementary Table S2**. Experimental linear retention index (*I^T^*) and resolution (*Rs*) data calculated on the three Es-columns tested on targeted chiral odorants.

| **Compound** | **Config.** | **Retention Index (*I^T^*)**  **(MeAc-TBDMSβCD)** | **Resolution *Rs***  **(MeAc-TBDMSβCD)** | **Retention Index (*I^T^*)**  **(MeMe-TBDMSβCD)** | **Resolution *Rs***  **(MeMe-TBDMSβCD)** | **Retention Index (*I^T^*)**  **AcAc-TBDMSβCD)** | **Resolution *Rs***  **(AcAc-TBDMSβCD)** |
| --- | --- | --- | --- | --- | --- | --- | --- |
| α-Pinene | *R* | 943 | 2.2 | 986 | 2.9 | 920 | 1.2 |
|  | *S* | 937 |  | 978 |  | 918 |  |
| Limonene | *R* | 1058 | 2.2 | 1068 | 5 | 1053 | NS |
|  | *S* | 1063 |  | 1057 |  | 1053 |  |
| Linalool | *R* | 1390 | 0.8 | 1207 | 3.9 | 1302 | 1.4 |
|  | *S* | 1392 |  | 1215 |  | 1305 |  |
| Ethyl-2-methylbutyrate | *R* | 928 | 5.5 | 881 | 2.9 | 919 | 2.7 |
|  | *S* | 940 |  | 885 |  | 924 |  |
| Filbertone | *R* | 1173 | 14.34 | 1052 | 9 | 1125 | 7.65 |
|  | *S* | 1210 |  | 1073 |  | 1147 |  |
| γ-Octalactone | *R* | 1849 | 12.7 | 1389 | 6.4 | 1716 | 4.6 |
|  | *S* | 1882 |  | 1404 |  | 1726 |  |
| γ-Hexalactone | *R* | 1742 | 14.5 | 1198 | 6 | 1637 | 15.7 |
|  | *S* | 1780 |  | 1210 |  | 1667 |  |
| γ-Heptalactone | *R* | 1774 | 14.6 | 1291 | 8.9 | 1661 | 4.2 |
|  | *S* | 1818 |  | 1310 |  | 1670 |  |
| γ-Pentalactone | *R* | 1751 | 15.3 | 1101 | 7.6 | 1663 | 21.4 |
|  | *S* | 1791 |  | 1117 |  | 1712 |  |
| γ-Nonalactone | *R* | 1941 | 12.6 | 1489 | 5.7 | 1789 | 3.9 |
|  | *S* | 1971 |  | 1503 |  | 1798 | on |
| δ-Hexalactone | X | 1850 | 5.6 | 1260 | NS | 1696 | 6.6 |
|  | Y | 1866 |  | 1260 |  | 1711 |  |

**Supplementary Table S3**. Targeted compounds detected in raw and roasted hazelnut samples: IUPAC names, trivial names, NIST Webbook link for spectral and retention data, Molecular formula and experimental *I^T^* on MeAc-TBDM-β-CD column. Chiral compounds whose enantiomer elution order is not yet known are reported with “X” for the first eluting and “Y” as the last eluting.

| **IUPAC names** | **Trivial names** | **NIST WEBBOOK URL** | **Molecular Formula** | **Exp. *I^T^*** | ***Ti* (*m/z*)** |
| --- | --- | --- | --- | --- | --- |
| (1R,2R,5S)-5-Isopropyl-2-methylbicyclo | (S)-(-)-Z-Sabinene hydrate | https://webbook.nist.gov/cgi/cbook.cgi?ID=U121973&Units=SI | C_10_H_18_O | 1337 | 71 |
| (1R,5R)-2,6,6-Trimethylbicyclo[3.1.1]hept-2-ene | (R)-(+)-α-Pinene | https://webbook.nist.gov/cgi/cbook.cgi?ID=C2437958&Units=SI | C_10_H_16_ | 943 | 93 |
| (1S,2S,5R)-5-Isopropyl-2-methylbicyclo-[3.1.0]-hexan-2-ol | (R)-(+)-(Z)-Sabinene hydrate | https://webbook.nist.gov/cgi/cbook.cgi?ID=U121973&Units=SI | C_10_H_18_O | 1326 | 71 |
| (1S,5S)-2,6,6-Trimethylbicyclo[3.1.1]hept-2-ene | (S)-(-)-α-Pinene | https://webbook.nist.gov/cgi/cbook.cgi?ID=C2437958&Units=SI | C_10_H_16_ | 937 | 93 |
| (2E,5R)-5-Methyl-2-hepten-4-one | (R)-Filbertone | https://webbook.nist.gov/cgi/cbook.cgi?ID=C102322838&Units=SI | C_8_H_14_O | 1173 | 98 |
| (2E,5S)-5-Methyl-2-hepten-4-one | (S)-Filbertone | https://webbook.nist.gov/cgi/cbook.cgi?ID=C102322838&Units=SI | C_8_H_14_O | 1210 | 98 |
| (2Z,5R)-5-Methyl-2-hepten-4-one |  | https://webbook.nist.gov/cgi/cbook.cgi?Name=5-Methyl-2-hepten-4-one&Units=SI | C_8_H_14_O | 1044 | 69 |
| (2Z,5S)-5-Methyl-2-hepten-4-one |  | https://webbook.nist.gov/cgi/cbook.cgi?Name=5-Methyl-2-hepten-4-one&Units=SI | C_8_H_14_O | 1050 | 69 |
| (3R)-3,7-Dimethylocta-1,6-dien-3-ol | (R)-Linalool | https://webbook.nist.gov/cgi/cbook.cgi?Name=Linalool&Units=SI | C_10_H_18_O | 1390 | 71 |
| (3S)-3,7-Dimethylocta-1,6-dien-3-ol | (S)-Linalool | https://webbook.nist.gov/cgi/cbook.cgi?Name=Linalool&Units=SI | C_10_H_18_O | 1392 | 71 |
| (4R)-4-Isopropenyl-1-methylcyclohexene | (R)-Limonene | https://webbook.nist.gov/cgi/cbook.cgi?ID=C138863&Units=SI | C_10_H_16_ | 1059 | 68 |
| (4S)-4-Isopropenyl-1-methylcyclohexene | (S)-Limonene | https://webbook.nist.gov/cgi/cbook.cgi?ID=C138863&Units=SI | C_10_H_16_ | 1063 | 68 |
| (5R)-2-Methyl-5-propan-2-ylcyclohexa-1,3-diene | (R)-(-)-α-Phellandrene | https://webbook.nist.gov/cgi/cbook.cgi?Name=Phellandrene&Units=SI | C_10_H_16_ | 1042 | 93 |
| (5S)-2-Methyl-5-propan-2-ylcyclohexa-1,3-diene | (S)-(+)- α-Phellandrene | https://webbook.nist.gov/cgi/cbook.cgi?Name=Phellandrene&Units=SI | C_10_H_16_ | 1046 | 93 |
| (E)-2-Butenal |  | https://webbook.nist.gov/cgi/cbook.cgi?Name=%28E%29-2-Butenal&Units=SI | C_4_H_6_O | 1094 | 70 |
| (E)-2-Decenal |  | https://webbook.nist.gov/cgi/cbook.cgi?Name=%28E%29-2-Decenal&Units=SI | C_10_H_18_O | 1626 | 70 |
| (E)-2-Heptenal |  | https://webbook.nist.gov/cgi/cbook.cgi?Name=%28E%29-2-Heptenal&Units=SI | C_7_H_12_O | 1367 | 83 |
| (E)-2-Hexenal |  | https://webbook.nist.gov/cgi/cbook.cgi?Name=%28E%29-2-Hexenal&Units=SI | C_6_H_10_O | 1263 | 83 |
| (E)-2-Methyl-2-butenal |  | https://webbook.nist.gov/cgi/cbook.cgi?Name=%28E%29-2-Methyl-2-butenal&Units=SI | C_5_H_8_O | 1215 | 55 |
| (E)-2-Nonenal |  | https://webbook.nist.gov/cgi/cbook.cgi?Name=%28E%29-2-Nonenal&Units=SI | C_9_H_16_O | 1543 | 70 |
| (E)-2-Octenal |  | https://webbook.nist.gov/cgi/cbook.cgi?Name=%28E%29-2-Octenal&Units=SI | C_8_H_14_O | 1456 | 70 |
| (E)-2-Undecenal |  | https://webbook.nist.gov/cgi/cbook.cgi?Name=%28E%29-2-Undecenal&Units=SI | C_11_H_20_O | 1712 | 83 |

| **IUPAC names** | **Trivial names** | **NIST WEBBOOK URL** | **Molecular Formula** | **Exp. *I^T^*** | ***Ti* (*m/z*)** |
| --- | --- | --- | --- | --- | --- |
| (E,E)-2,4-Decadienal |  | https://webbook.nist.gov/cgi/cbook.cgi?Name=%28E%2CE%29-2%2C4-Decadienal&Units=SI | C_10_H_16_O | 1701 | 81 |
| (E,E)-2,4-Nonadienal |  | https://webbook.nist.gov/cgi/cbook.cgi?Name=%28E%2CE%29-2%2C4-Nonadienal&Units=SI | C_9_H_14_O | 1615 | 81 |
| (R)-5-Butyldihydro-2(3H)-furanone | (R)-γ-Octalactone | https://webbook.nist.gov/cgi/cbook.cgi?Name=%CE%B3-Octalactone&Units=SI | C_8_H_14_O_2_ | 1849 | 85 |
| (R)-5-Ethyldihydro-2(3H)-furanone | (R)-γ-Hexalactone | https://webbook.nist.gov/cgi/cbook.cgi?Name=%CE%B3-Hexalactone&Units=SI | C_6_H_10_O_2_ | 1742 | 85 |
| (R)-5-Methyl-2(5H)-furanone | (R)-β-angelica lactone | https://webbook.nist.gov/cgi/cbook.cgi?Name=%CE%B2-angelica+lactone&Units=SI | C_5_H_6_O_2_ | 1787 | 55 |
| (R)-Dihydro-5-methyl-2(3H)-furanone | (R)-γ-Pentalactone | https://webbook.nist.gov/cgi/cbook.cgi?ID=C108292&Units=SI | C_5_H_8_O_2_ | 1751 | 56 |
| (R)-Dihydro-5-pentyl-2(3H)-furanone | (R)-γ-Nonalactone | https://webbook.nist.gov/cgi/cbook.cgi?Name=%CE%B3-Nonalactone&Units=SI | C_9_H_16_O_2_ | 1941 | 85 |
| (R)-Dihydro-5-propyl- 2(3H)-furanone | (R)-γ-Heptalactone | https://webbook.nist.gov/cgi/cbook.cgi?Name=%CE%B3-Heptalactone&Units=SI | C_7_H_12_O_2_ | 1774 | 85 |
| (R)-Ethyl-2-methylbutanoate | (R)-Ethyl-2-methylbutyrate | https://webbook.nist.gov/cgi/cbook.cgi?Name=Ethyl-2-methylbutyrate&Units=SI | C_7_H_14_O_2_ | 928 | 102 |
| (S)-5-Butyldihydro-2(3H)-furanone | (S)-γ-Octalactone | https://webbook.nist.gov/cgi/cbook.cgi?Name=%CE%B3-Octalactone&Units=SI | C_8_H_14_O_2_ | 1882 | 85 |
| (S)-5-Ethyldihydro- 2(3H)-furanone | (S)-γ-Hexalactone | https://webbook.nist.gov/cgi/cbook.cgi?Name=%CE%B3-Hexalactone&Units=SI | C_6_H_10_O_2_ | 1780 | 85 |
| (S)-5-Methyl-2(5H)-furanone | (S)-β-angelica lactone | https://webbook.nist.gov/cgi/cbook.cgi?Name=%CE%B2-angelica+lactone&Units=SI | C_5_H_6_O_2_ | 1865 | 55 |
| (S)-Dihydro-5-methyl-2(3H)-furanone | (S)-γ-Pentalactone | https://webbook.nist.gov/cgi/cbook.cgi?ID=C108292&Units=SI | C_5_H_8_O_2_ | 1791 | 56 |
| (S)-Dihydro-5-pentyl-2(3H)-furanone | (S)-γ -Nonalactone | https://webbook.nist.gov/cgi/cbook.cgi?Name=%CE%B3-Nonalactone&Units=SI | C_9_H_16_O_2_ | 1971 | 85 |
| (S)-Ethyl-2-methylbutanoate | (S)-Ethyl-2-methylbutyrate | https://webbook.nist.gov/cgi/cbook.cgi?Name=Ethyl-2-methylbutyrate&Units=SI | C_7_H_14_O_2_ | 940 | 102 |
| (X) (X)-2,3-Butanediol |  | https://webbook.nist.gov/cgi/cbook.cgi?ID=C513893&Units=SI | C_4_H_10_O_2_ | 1455 | 45 |
| (X) (X)-2,4-Pentanediol |  | https://webbook.nist.gov/cgi/cbook.cgi?Name=2%2C4-Pentanediol&Units=SI | C_5_H_12_O_2_ | 1531 | 45 |
| (X) (Y)-2,3-Butanediol |  | https://webbook.nist.gov/cgi/cbook.cgi?ID=C513893&Units=SI | C_4_H_10_O_2_ | 1478 | 45 |
| (X) (Y)-2,4-Pentanediol |  | https://webbook.nist.gov/cgi/cbook.cgi?Name=2%2C4-Pentanediol&Units=SI | C_5_H_12_O_2_ | 1527 | 45 |
| (X)-2,3-Dihydro-3,5-dihydroxy-6-methyl-4H-pyran-4-one |  | https://webbook.nist.gov/cgi/cbook.cgi?Name=2%2C3-Dihydro-3%2C5-dihydroxy-6-methyl-4H-pyran-4-one&Units=SI | C_6_H_8_O_4_ | 1730 | 144 |
| (X)-2-Pentanol |  | https://webbook.nist.gov/cgi/cbook.cgi?Name=2-Pentanol&Units=SI | C_5_H_12_O | 1024 | 45 |
| (X)-3-Hydroxy-2-butanone |  | https://webbook.nist.gov/cgi/cbook.cgi?ID=C513860&Units=SI | C_4_H_8_O_2_ | 1376 | 88 |
| (X)-3-Methyl-4-Heptanone |  | https://webbook.nist.gov/cgi/cbook.cgi?Name=3-Methyl-4-Heptanone&Units=SI | C_8_H_16_O | 1042 | 57 |
| (X)-3-Penten-2-one |  | https://webbook.nist.gov/cgi/cbook.cgi?Name=3-Penten-2-one&Units=SI | C_5_H_8_O | 1215 | 84 |

| **IUPAC names** | **Trivial names** | **NIST WEBBOOK URL** | **Molecular Formula** | **Exp. *I^T^*** | ***Ti* (*m/z*)** |
| --- | --- | --- | --- | --- | --- |
| (Y) (X)-2,3-Butanediol |  | https://webbook.nist.gov/cgi/cbook.cgi?ID=C513893&Units=SI | C_4_H_10_O_2_ | 1433 | 45 |
| (Y) (X)-2,4-Pentanediol |  | https://webbook.nist.gov/cgi/cbook.cgi?Name=2%2C4-Pentanediol&Units=SI | C_5_H_12_O_2_ | 1536 | 45 |
| (Y)-2,3-Dihydro-3,5-dihydroxy-6-methyl-4H-pyran-4-one |  | https://webbook.nist.gov/cgi/cbook.cgi?Name=2%2C3-Dihydro-3%2C5-dihydroxy-6-methyl-4H-pyran-4-one&Units=SI | C_6_H_8_O_4_ | 1754 | 144 |
| (Y)-2-Pentanol |  | https://webbook.nist.gov/cgi/cbook.cgi?Name=2-Pentanol&Units=SI | C_5_H_12_O | 1027 | 45 |
| (Y)-3-Hydroxy-2-butanone |  | https://webbook.nist.gov/cgi/cbook.cgi?ID=C513860&Units=SI | C_4_H_8_O_2_ | 1416 | 88 |
| (Y)-3-Methyl-4-Heptanone |  | https://webbook.nist.gov/cgi/cbook.cgi?Name=3-Methyl-4-Heptanone&Units=SI | C_8_H_16_O | 1055 | 57 |
| (Y)-3-Penten-2-one |  | https://webbook.nist.gov/cgi/cbook.cgi?Name=3-Penten-2-one&Units=SI | C_5_H_8_O | 1216 | 84 |
| 1,3-Cyclopentadiene |  | https://webbook.nist.gov/cgi/cbook.cgi?Name=1%2C3-Cyclopentadiene&Units=SI | C_5_H_6_ | <900 | 66 |
| 1-Decene |  | https://webbook.nist.gov/cgi/cbook.cgi?Name=1-Decene&Units=SI | C_10_H_20_ | 1011 | 70 |
| 1-Ethyl-1H-pyrrole |  | https://webbook.nist.gov/cgi/cbook.cgi?Name=1-Ethyl-1H-pyrrole&Units=SI | C_6_H_9_N | 1198 | 80 |
| 1-Heptanol |  | https://webbook.nist.gov/cgi/cbook.cgi?Name=1-Heptanol&Units=SI | C_7_H_16_O | 1230 | 70 |
| 1-Hexanol |  | https://webbook.nist.gov/cgi/cbook.cgi?Name=1-Hexanol&Units=SI | C_6_H_14_O | 1146 | 56 |
| 1-Hydroxy-2-propanone |  | https://webbook.nist.gov/cgi/cbook.cgi?Name=1-Hydroxy-2-propanone&Units=SI | C_3_H_6_O_2_ | 1295 | 43 |
| 1-Isopropyl-4-methylbicyclo[3.1.0]hex-3-ene | α-Thujene | https://webbook.nist.gov/cgi/cbook.cgi?Name=%CE%B1-Thujene&Units=SI | C_10_H_16_ | 941 | 93 |
| 1-Methyl-2-pyrrolidinone |  | https://webbook.nist.gov/cgi/cbook.cgi?Name=1-Methyl-2-pyrrolidinone&Units=SI | C_5_H_9_NO | 1705 | 99 |
| 1-Nonene |  | https://webbook.nist.gov/cgi/cbook.cgi?Name=1-Nonene&Units=SI | C_9_H_18_ | 912 | 56 |
| 1-Octanol |  | https://webbook.nist.gov/cgi/cbook.cgi?Name=1-Octanol&Units=SI | C_8_H_18_O | 1316 | 56 |
| 1-Pentanol |  | https://webbook.nist.gov/cgi/cbook.cgi?Name=1-Pentanol&Units=SI | C_5_H_12_O | 1058 | 55 |
| 2(5H)-Furanone | γ -Crotonolactone | https://webbook.nist.gov/cgi/cbook.cgi?Name=2%285H%29-Furanone&Units=SI | C_4_H_4_O_2_ | 1853 |  |
| 2,3,5-Trimethylfuran |  | https://webbook.nist.gov/cgi/cbook.cgi?Name=2%2C3%2C5-Trimethylfuran&Units=SI | C_7_H_10_O | <900 | 110 |
| 2,3-Dimethylpyrazine |  | https://webbook.nist.gov/cgi/cbook.cgi?Name=2%2C3-Dimethylpyrazine&Units=SI | C_6_H_8_N_2_ | 1210 | 108 |
| 2,5-Diethylpyrazine |  | https://webbook.nist.gov/cgi/cbook.cgi?Name=2%2C5-Diethylpyrazine&Units=SI | C_8_H_12_N_2_ | 1304 | 121 |
| 2,5-Dimethyl-4-hydroxy-3(2H)-furanone | Furaneol | https://webbook.nist.gov/cgi/cbook.cgi?Name=2%2C5-Dimethyl-4-hydroxy-3%282H%29-furanone&Units=SI | C_6_H_8_O_3_ | 1638 | 128 |
| 2,5-Dimethylfuran |  | https://webbook.nist.gov/cgi/cbook.cgi?Name=2%2C5-Dimethylfuran&Units=SI | C_6_H_8_O | <900 | 96 |

| **IUPAC names** | **Trivial names** | **NIST WEBBOOK URL** | **Molecular Formula** | **Exp. *I^T^*** | ***Ti* (*m/z*)** |
| --- | --- | --- | --- | --- | --- |
| 2,5-Dimethylpyrazine |  | https://webbook.nist.gov/cgi/cbook.cgi?Name=2%2C5-Dimethylpyrazine&Units=SI | C_6_H_8_N_2_ | 1120 | 108 |
| 2,6-Dimethylpyrazine |  | https://webbook.nist.gov/cgi/cbook.cgi?Name=2%2C6-Dimethylpyrazine&Units=SI | C_6_H_8_N_2_ | 1149 | 108 |
| 2-Acetylpyridine |  | https://webbook.nist.gov/cgi/cbook.cgi?Name=2-Acetylpyridine&Units=SI | C_7_H_7_NO | 1407 | 79 |
| 2-Butanone |  | https://webbook.nist.gov/cgi/cbook.cgi?Name=2-Carboxaldehyde-1H-pyrrole&Units=SI | C_5_H_5_NO | 1026 | 43 |
| 2-Carboxaldehyde-1H-pyrrole | Formyl pyrrole | https://webbook.nist.gov/cgi/cbook.cgi?Name=2-Carboxaldehyde-1H-pyrrole&Units=SI | C_5_H_5_NO | 1581 | 95 |
| 2-Cyclopentene-1,4-dione |  | https://webbook.nist.gov/cgi/cbook.cgi?Name=2-Cyclopentene-1%2C4-dione&Units=SI | C_5_H_4_O_2_ | 1578 | 96 |
| 2-Decanone |  | https://webbook.nist.gov/cgi/cbook.cgi?Name=2-Decanone&Units=SI | C_10_H_20_O | 1435 | 58 |
| 2-Ethyl-5-methylpyrazine |  | https://webbook.nist.gov/cgi/cbook.cgi?Name=2-Ethyl-5-methylpyrazine&Units=SI | C_7_H_10_N_2_ | 1225 | 122 |
| 2-Ethyl-6-methylpyrazine |  | https://webbook.nist.gov/cgi/cbook.cgi?Name=2-Ethyl-6-methylpyrazine&Units=SI | C_7_H_10_N_2_ | 1200 | 121 |
| 2-Furancarboxaldehyde | Furfural | https://webbook.nist.gov/cgi/cbook.cgi?Name=2-Furancarboxaldehyde&Units=SI | C_5_H_4_O_2_ | 1364 | 96 |
| 2-Furanmethanol |  | https://webbook.nist.gov/cgi/cbook.cgi?Name=2-Furanmethanol&Units=SI | C_5_H_6_O_2_ | 1276 | 98 |
| 1-(2-Furyl)-2-hydroxyethanone | 2-Furylhydroxymethyl ketone | https://webbook.nist.gov/cgi/cbook.cgi?Name=1-%282-Furyl%29-2-hydroxyethanone&Units=SI | C_6_H_6_O_3_ | 1644 | 95 |
| 2-Heptanone |  | https://webbook.nist.gov/cgi/cbook.cgi?Name=2-Heptanone&Units=SI | C_7_H_14_O | 1165 | 58 |
| 2-Methyl-1H-pyrrole |  | https://webbook.nist.gov/cgi/cbook.cgi?Name=2-Methyl-1H-pyrrole&Units=SI | C_5_H_7_N | 1229 | 121 |
| 2-Methylbutanal |  | https://webbook.nist.gov/cgi/cbook.cgi?Name=2-Methylbutanal&Units=SI | C_5_H_10_O | <900 | 41 |
| 2-Methylfuran |  | https://webbook.nist.gov/cgi/cbook.cgi?Name=2-Methylfuran&Units=SI | C_5_H_6_O | <900 | 82 |
| 2-Methylpentane |  | https://webbook.nist.gov/cgi/cbook.cgi?Name=2-Methylpentane&Units=SI | C_6_H_14_ | <900 | 43 |
| 2-Methylpropanal |  | https://webbook.nist.gov/cgi/cbook.cgi?Name=2-Methylpropanal&Units=SI | C_4_H_8_O | 991 | 43 |
| 2-Nonanone |  | https://webbook.nist.gov/cgi/cbook.cgi?Name=2-Nonanone&Units=SI | C_9_H_18_O | 1347 | 58 |
| 2-Octanone |  | https://webbook.nist.gov/cgi/cbook.cgi?Name=2-Octanone&Units=SI | C_8_H_16_O | 1259 | 58 |
| 2-Pentanone | Ethyl acetone | https://webbook.nist.gov/cgi/cbook.cgi?Name=2-Pentanone&Units=SI | C_5_H_10_O | 1065 | 43 |
| 2-Phenyl-2-butenal |  | https://webbook.nist.gov/cgi/cbook.cgi?Name=2-Phenyl-2-butenal&Units=SI | C_10_H_10_O | 1493 | 117 |
| 2-Propanone | Acetone | https://webbook.nist.gov/cgi/cbook.cgi?Name=2-Propanone&Units=SI | C_3_H_6_O | 919 | 43 |
| 2-Pyrrolidinone |  | https://webbook.nist.gov/cgi/cbook.cgi?Name=2-Pyrrolidinone&Units=SI | C_4_H_7_NO | 1958 | 42 |
| 2-Undecanone |  | https://webbook.nist.gov/cgi/cbook.cgi?Name=2-Undecanone&Units=SI | C_11_H_22_O | 1525 | 71 |

| **IUPAC names** | **Trivial names** | **NIST WEBBOOK URL** | **Molecular Formula** | **Exp. *I^T^*** | ***Ti* (*m/z*)** |
| --- | --- | --- | --- | --- | --- |
| 2-Vinylfuran |  | https://webbook.nist.gov/cgi/cbook.cgi?Name=2-Vinylfuran&Units=SI | C_6_H_6_O | <900 | 94 |
| 3,5,5-Trimethyl-2(5H)-furanone |  | https://webbook.nist.gov/cgi/cbook.cgi?Name=3%2C5%2C5-Trimethyl-2%285H%29-furanone&Units=SI | C_7_H_10_O_2_ | 1933 |  |
| 3-Ethyl-2,5-dimethylpyrazine |  | https://webbook.nist.gov/cgi/cbook.cgi?Name=3-Ethyl-2%2C5-dimethylpyrazine&Units=SI | C_8_H_12_N_2_ | 1234 | 135 |
| 3-Hexanone |  | https://webbook.nist.gov/cgi/cbook.cgi?Name=3-Hexanone&Units=SI | C_6_H_12_O | 1026 | 71 |
| 3-Hydroxy-2-methyl-4H-pyran-4-one |  | https://webbook.nist.gov/cgi/cbook.cgi?Name=3-Hydroxy-2-methyl-4H-pyran-4-one&Units=SI | C_6_H_6_O_3_ | 1720 | 126 |
| 3-Methyl-1H-pyrrole |  | https://webbook.nist.gov/cgi/cbook.cgi?Name=3-Methyl-1H-pyrrole&Units=SI | C_5_H_7_N | 1296 | 80 |
| 3-Methyl-2(5H)-furanone | α-Methyl-γ-crotonolactone | https://webbook.nist.gov/cgi/cbook.cgi?Name=3-Methyl-2%285H%29-furanone&Units=SI | C_5_H_6_O_2_ | 1816 | 98 |
| 3-Methyl-2-cyclopenten-1-one |  | https://webbook.nist.gov/cgi/cbook.cgi?Name=3-Methyl-2-cyclopenten-1-one&Units=SI | C_6_H_8_O | 1630 | 96 |
| 3-Methyl-2-pentanone |  | https://webbook.nist.gov/cgi/cbook.cgi?Name=3-Methyl-2-pentanone&Units=SI | C_6_H_12_O | 1110 | 43 |
| 3-Methylbutanal |  | https://webbook.nist.gov/cgi/cbook.cgi?Name=3-Methylbutanal&Units=SI | C_5_H_10_O | 1031 | 58 |
| 3-Methylphenol |  | https://webbook.nist.gov/cgi/cbook.cgi?Name=3-Methylphenol&Units=SI | C_7_H_8_O | 935 | 108 |
| 3-Octen-2-one |  | https://webbook.nist.gov/cgi/cbook.cgi?Name=3-Octen-2-one&Units=SI | C_8_H_14_O | 1419 | 111 |
| 3-Penten-2-ol |  | https://webbook.nist.gov/cgi/cbook.cgi?Name=3-Penten-2-ol&Units=SI | C_5_H_10_O | 998 | 71 |
| 3-Undecanone |  | https://webbook.nist.gov/cgi/cbook.cgi?Name=3-Undecanone&Units=SI | C_11_H_22_O | 1462 | 72 |
| 4-Heptanone |  | https://webbook.nist.gov/cgi/cbook.cgi?Name=4-Heptanone&Units=SI | C_7_H_14_O | 1055 | 71 |
| 4-Methyl-2(5H)-furanone |  | https://webbook.nist.gov/cgi/cbook.cgi?Name=4-Methyl-2%285H%29-furanone&Units=SI | C_5_H_6_O_2_ | 2075 | 98 |
| 4-methylene-1-(1-methylethyl) bicyclo[3.1.0]hexane | Sabinene | https://webbook.nist.gov/cgi/cbook.cgi?Name=Sabinene&Units=SI | C_10_H_16_ | 1098 | 93 |
| 4-Octen-3-one |  | https://webbook.nist.gov/cgi/cbook.cgi?Name=4-Octen-3-one&Units=SI | C_8_H_14_O | 1227 | 97 |
| 5,6-Dihydro-2H-pyran-2-one |  | https://webbook.nist.gov/cgi/cbook.cgi?Name=5%2C6-Dihydro-2H-pyran-2-one&Units=SI | C_5_H_6_O_2_ | 2014 | 98 |
| 5,6-Dihydro-6-pentyl-2H-pyran-2-one | C-10-Massoia lactone | https://webbook.nist.gov/cgi/cbook.cgi?Name=5%2C6-Dihydro-6-pentyl-2H-pyran-2-one&Units=SI | C_10_H_16_O_2_ | 1905 | 55 |
| 5-Ethyl-2,3-dimethylpyrazine |  | https://webbook.nist.gov/cgi/cbook.cgi?Name=5-Ethyl-2%2C3-dimethylpyrazine&Units=SI | C_8_H_12_N_2_ | 1293 | 135 |
| 5-Hydroxymethyl-2-furancarboxaldehyde | 5-(hydroxymethyl)-furfural | https://webbook.nist.gov/cgi/cbook.cgi?Name=5-Hydroxymethyl-2-furancarboxaldehyde&Units=SI | C_6_H_6_O_3_ | 1931 | 126 |
| 6-Methyl-5-hepten-2-one |  | https://webbook.nist.gov/cgi/cbook.cgi?Name=6-Methyl-5-hepten-2-one&Units=SI | C_8_H_14_O | 1218 | 108 |
| Benzaldehyde |  | https://webbook.nist.gov/cgi/cbook.cgi?Name=Benzaldehyde&Units=SI | C_7_H_6_O | 940 | 106 |
| Butanal |  | https://webbook.nist.gov/cgi/cbook.cgi?Name=Butanal&Units=SI | C_4_H_8_O | 977 | 72 |

| **IUPAC names** | **Trivial names** | **NIST WEBBOOK URL** | **Molecular Formula** | **Exp. *I^T^*** | ***Ti* (*m/z*)** |
| --- | --- | --- | --- | --- | --- |
| Decanal |  | https://webbook.nist.gov/cgi/cbook.cgi?Name=Decanal&Units=SI | C_10_H_20_O | 1448 | 70 |
| Decane |  | https://webbook.nist.gov/cgi/cbook.cgi?Name=Decane&Units=SI | C_10_H_22_ | 995 | 57 |
| Dihydro-2(3H)-furanone | γ -Butyrolactone | https://webbook.nist.gov/cgi/cbook.cgi?Name=%CE%B3+-Butyrolactone&Units=SI | C_4_H_6_O_2_ | 1807 | 42 |
| Dihydro-3-hydroxy-4,4-dimethyl-2(3H)-furanone | Pantolactone | https://webbook.nist.gov/cgi/cbook.cgi?Name=Pantolactone&Units=SI | C_6_H_10_O_3_ | 1998 | 71 |
| Dodecane |  | https://webbook.nist.gov/cgi/cbook.cgi?Name=Dodecane&Units=SI | C_12_H_26_ | 1200 | 57 |
| Ethanal | Acetaldehyde | https://webbook.nist.gov/cgi/cbook.cgi?Name=Ethanal&Units=SI | C_2_H_4_O | <900 | 44 |
| Ethanoic acid | Acetic acid | https://webbook.nist.gov/cgi/cbook.cgi?Name=Ethanoic+acid&Units=SI | C_2_H_4_O_2_ | 1144 | 60 |
| Ethanol |  | https://webbook.nist.gov/cgi/cbook.cgi?Name=Ethanol&Units=SI | C_2_H_6_O | <900 | 31 |
| Ethenyl hexanoate | Vinyl caproate | https://webbook.nist.gov/cgi/cbook.cgi?Name=Vinyl+caproate&Units=SI | C_8_H_14_O_2_ | 1541 | 71 |
| Ethyl acetate |  | https://webbook.nist.gov/cgi/cbook.cgi?Name=Ethyl+acetate&Units=SI | C_4_H_8_O_2_ | 1517 | 43 |
| Ethyl octanoate |  | https://webbook.nist.gov/cgi/cbook.cgi?Name=Ethyl+octanoate&Units=SI | C_10_H_20_O_2_ | 1301 | 88 |
| Ethylpyrazine |  | https://webbook.nist.gov/cgi/cbook.cgi?Name=Ethylpyrazine&Units=SI | C_6_H_8_N_2_ | 1154 | 107 |
| Heptanal |  | https://webbook.nist.gov/cgi/cbook.cgi?Name=Heptanal&Units=SI | C_7_H_14_O | 1183 | 70 |
| Heptane |  | https://webbook.nist.gov/cgi/cbook.cgi?Name=Heptane&Units=SI | C_7_H_16_ | <900 | 43 |
| Hexanal | Caproaldehyde | https://webbook.nist.gov/cgi/cbook.cgi?Name=Hexanal&Units=SI | C_6_H_12_O | 1083 | 56 |
| Hexane |  | https://webbook.nist.gov/cgi/cbook.cgi?Name=Hexane&Units=SI | C_6_H_14_ | <900 | 57 |
| Hexanoic acid |  | https://webbook.nist.gov/cgi/cbook.cgi?Name=Hexanoic+acid&Units=SI | C_6_H_12_O_2_ | 1432 | 60 |
| Methyl ethanoate | Methyl acetate | https://webbook.nist.gov/cgi/cbook.cgi?Name=Methyl+ethanoate&Units=SI | C_3_H_6_O_2_ | <900 | 43 |
| Methyl nonanoate |  | https://webbook.nist.gov/cgi/cbook.cgi?Name=Methyl+nonanoate&Units=SI | C_10_H_20_O_2_ | 1340 | 74 |
| Methyl octanoate |  | https://webbook.nist.gov/cgi/cbook.cgi?Name=Methyl+octanoate&Units=SI | C_9_H_18_O_2_ | 1246 | 74 |
| Methyl-(1-methylethyl)-benzene | Cymene | https://webbook.nist.gov/cgi/cbook.cgi?Name=Methyl-%281-methylethyl%29-benzene&Units=SI | C_10_H_14_ | 1112 | 119 |
| Methyl-2-propenoate |  | https://webbook.nist.gov/cgi/cbook.cgi?Name=Methyl-2-propenoate&Units=SI | C_4_H_6_O_2_ | <900 | 55 |
| Methyl-2-pyridinecarboxylate |  | https://webbook.nist.gov/cgi/cbook.cgi?Name=Methyl-2-pyridinecarboxylate&Units=SI | C_7_H_7_NO_2_ | 1650 | 79 |
| Methylpyrazine |  | https://webbook.nist.gov/cgi/cbook.cgi?Name=Methylpyrazine&Units=SI | C_5_H_6_N_2_ | 1088 | 94 |
| Nonanal |  | https://webbook.nist.gov/cgi/cbook.cgi?Name=Nonanal&Units=SI | C_9_H_18_O | 1356 | 57 |
| Nonane |  | https://webbook.nist.gov/cgi/cbook.cgi?Name=Nonane&Units=SI | C_9_H_20_ | 899 | 57 |

| **IUPAC names** | **Trivial names** | **NIST WEBBOOK URL** | **Molecular Formula** | **Exp. *I^T^*** | ***Ti* (*m/z*)** |
| --- | --- | --- | --- | --- | --- |
| Octanal |  | https://webbook.nist.gov/cgi/cbook.cgi?Name=Octanal&Units=SI | C_8_H_16_O | 1268 | 84 |
| Octane |  | https://webbook.nist.gov/cgi/cbook.cgi?Name=Octane&Units=SI | C_8_H_18_ | <900 | 43 |
| Octanenitrile |  | https://webbook.nist.gov/cgi/cbook.cgi?Name=Octanenitrile&Units=SI | C_8_H_15_N | 1536 | 82 |
| Pentanal | Valeraldehyde | https://webbook.nist.gov/cgi/cbook.cgi?Name=Pentanal&Units=SI | C_5_H_10_O | 1027 | 44 |
| Phenylformamide |  | https://webbook.nist.gov/cgi/cbook.cgi?Name=Phenylformamide&Units=SI | C_7_H_7_NO | <900 | 121 |
| Pyrazinamide |  | https://webbook.nist.gov/cgi/cbook.cgi?Name=Pyrazinamide&Units=SI | C_5_H_5_N_3_O | 1640 | 123 |
| Pyrazine |  | https://webbook.nist.gov/cgi/cbook.cgi?Name=Pyrazine&Units=SI | C_4_H_4_N_2_ | 1016 | 80 |
| Pyrrole |  | https://webbook.nist.gov/cgi/cbook.cgi?Name=Pyrrole&Units=SI | C_4_H_5_N | 1239 | 67 |
| Tetrahydro-2H-pyran-2-one | δ-Valerolactone | https://webbook.nist.gov/cgi/cbook.cgi?Name=Tetrahydro-2H-pyran-2-one&Units=SI | C_5_H_8_O_2_ | 1975 | 100 |
| (X) Tetrahydro-6-methyl-2H-pyran-2-one | δ-Hexalactone | https://webbook.nist.gov/cgi/cbook.cgi?Name=%CE%B4-Hexalactone&Units=SI | C_6_H_10_O_2_ | 1850 | 70 |
| (X) Tetrahydro-6-propyl-2H-Pyran-2-one | δ-Octalactone | https://webbook.nist.gov/cgi/cbook.cgi?Name=%CE%B4-Octalactone&Units=SI | C_8_H_14_O_2_ | 1842 | 99 |
| Trimethylpyrazine |  | https://webbook.nist.gov/cgi/cbook.cgi?Name=Trimethylpyrazine&Units=SI | C_7_H_10_N_2_ | 1235 | 122 |

**Supplementary Table S4**. Odor quality and odor threshold (in oil; air or water media are included when not available in oil) for enantiomers of hazelnut chiral odorants.

| **Compound** | **Configuration** | **Odor quality** | **Odor threshold in oil (mg/L)** |
| --- | --- | --- | --- |
| α-Pinene | *R* | *Harsh, terpene-like, minty* | 2,100 (air) |
|  | *S* | *Harsh, terpene-like, coniferous* | 3,300 (air) |
| Limonene | *R* | *Fresh, citrus orange-like* | 200 |
|  | *S* | *Harsh, terpene-like* | 500 |
| Linalool | *R* | *Floral, woody* | 8*10^-4^ |
|  | *S* | *Sweet, floral* | 7.4*10^-3^ |
| Ethyl-2-methylbutyrate | *R* | *Fruity, apple-like* | \ |
|  | *S* | *Fresh, fruity* | 6*10^-6^ (water) |
| Filbertone | *R* | *Hazelnut, soft, buttery* | 3.8*10^-3^ |
|  | *S* | *Hazelnut, metallic, fatty* | \ |
| α-Phellandrene | *R* | *Terpene-like, medicinal* | 0.5 (water) |
|  | *S* | *Weed-like, dill-like* | 0.2 (water) |
| γ-Octalactone | *R* | *Spicy, green* | 0.3 (water) |
|  | *S* | *Sweet, coconut-like* | 0.13 (water) |
| γ-Hexalactone | *R* | *Sweet, creamy, coconut-like* | \ |
|  | *S* | *Faint, sweet, fatty* | \ |
| γ-Heptalactone | *R* | *Sweet, herbaceous* | 0.7 (water) |
|  | *S* | *Fatty, coconut-like* | 0.89 (water) |
| γ-Pentalactone | *R* | *\* | 20 |
|  | *S* | *\* | \ |
| γ-Nonalactone | *R* | *Sweet, coconut-like* | 0.22 (water) |
|  | *S* | *Fatty, moldy* | 0.15 (water) |

**Supplementary Table S5**. Averaged response data for the targeted analytes over the 14 hazelnut batches analyzed.

Data are provided as excel file.

**Figures**

**Figure S1**. Chromatographic profiles of (E)- and (Z)-5-methyl-2-hepten-4-one on the three investigated chiral columns (i.e. (a) MeMe-TBDMS β-CD, (b) AcAc-TBDMS-β-CD, and (c) MeAc-TBDMS-β-CD).

**Figure S2:** PLS-DA of *Tonda Gentile Trilobata* harvested in Italy (IT-T) and in Georgia (GE-T) for raw (A) and roasted (B) samples.

**Figure S3.** Variable importance in the projection score (VIPs) resulting from the PLS-DA model on raw samples. Discrimination for cultivars along the first two components (t1 and t2 - A1 and A2) and discrimination based on origin (B).

**Figure S4.** PLS-DA for roasted samples of *Tonda Gentile Trilobata*, *Tonda Gentile Romana* and *Anakliuri.* Model discriminating cultivars (A) and origins (B) in roasted samples.
